# Effect of a Peer Comparison and Educational Intervention on Medical Test Conversation Quality: A Randomized Clinical Trial

**DOI:** 10.1001/jamanetworkopen.2023.42464

**Published:** 2023-11-09

**Authors:** Ishani Ganguli, Kathleen L. Mulligan, Emma D. Chant, Stuart Lipsitz, Leigh Simmons, Karen Sepucha, Robert S. Rudin

**Affiliations:** 1 Harvard Medical School, Boston, Massachusetts; 2 Division of General Internal Medicine and Primary Care, Brigham and Women’s Hospital, Boston, Massachusetts; 3 Frank H. Netter MD School of Medicine at Quinnipiac University, North Haven, Connecticut; 4 Hackensack Meridian School of Medicine, Nutley, New Jersey; 5 Division of General Internal Medicine, Massachusetts General Hospital, Boston, Massachusetts; 6 Health Care Division, RAND Corporation, Boston, Massachusetts

## Abstract

**Importance:**

Medical test overuse and resulting care cascades represent a costly, intractable problem associated with inadequate patient-clinician communication. One possible solution with potential for broader benefits is priming routine, high-quality medical test conversations.

**Objective:**

To assess if a peer comparison and educational intervention for physicians and patients improved medical test conversations during annual visits.

**Design, Setting, and Participants:**

Randomized clinical trial and qualitative evaluation at an academic medical center conducted May 2021 to October 2022. Twenty primary care physicians (PCPs) were matched-pair randomized. For each physician, at least 10 patients with scheduled visits were enrolled. Data were analyzed from December 2022 to September 2023.

**Interventions:**

In the intervention group, physicians received previsit emails that compared their low-value testing rates with those of peer PCPs and included point-of-care–accessible guidance on medical testing; patients received previsit educational materials via email and text message. Control group physicians and patients received general previsit preparation tips.

**Main outcomes and measures:**

The primary patient outcome was the Shared Decision-Making Process survey (SDMP) score. Secondary patient outcomes included medical test knowledge and presence of test conversation. Outcomes were compared using linear regression models adjusted for patient age, gender, race and ethnicity, and education. Poststudy interviews with intervention group physicians and patients were also conducted.

**Results:**

There were 166 intervention group patients and 148 control group patients (mean [SD] patient age, 50.2 [15.3] years; 210 [66.9%] female; 246 [78.3%] non-Hispanic White). Most patients discussed at least 1 test with their physician (95.4% for intervention group; 98.3% for control group; difference, −2.9 percentage points; 95% CI, −7.0 to 1.2 percentage points). There were no statistically significant differences in SDMP scores (2.11 out of 4 for intervention group; 1.97 for control group; difference, 0.14; 95% CI, −0.25 to 0.54) and knowledge scores (2.74 vs 2.54 out of 4; difference, 0.19; 95% CI, −0.05 to 0.43). In poststudy interviews with 3 physicians and 16 patients, some physicians said the emails helped them reexamine their testing approach while others noted competing demands. Most patients said they trusted their physicians’ advice even when inconsistent with educational materials.

**Conclusions and Relevance:**

In this randomized clinical trial of a physician-facing and patient-facing peer comparison and educational intervention, there was no significant improvement in medical test conversation quality during annual visits. These results suggest that future interventions to improve conversations and reduce overuse and cascades should further address physician adoption barriers and leverage patient-clinician relationships.

**Trial Registration:**

ClinicalTrials.gov Identifier: NCT04902664

## Key Points

**Question:**

Did a peer comparison and educational intervention improve shared decision-making conversations about medical testing during annual physicals?

**Findings:**

In this randomized clinical trial of 20 primary care physicians and 314 patients, the intervention did not significantly improve conversations (Shared Decision-Making Process survey score 2.11 for intervention group vs 1.97 for control). In poststudy interviews, physicians noted competing demands during visits; patients trusted their physicians’ testing decisions even when inconsistent with educational materials.

**Meaning:**

Results of this initial trial suggest that future interventions to improve medical test conversations and reduce test overuse might focus on easing physician adoption barriers and leveraging patient-clinician relationships.

## Teaser

This randomized clinical trial evaluates the effects of a peer compairson and education intervention on medical test conversation quality between primary care physicians and patients during annual visits.

## Introduction

Medical test overuse is a costly and intractable problem.^1,2^ Low-value tests such as screening electrocardiograms in healthy adults or early imaging for uncomplicated low back pain can cause direct and cascading patient and clinician harms, contributing to an estimated $100 billion in wasteful health care spending on low-value care annually in the US.^3,4,5,6^ Prior efforts to reduce this overuse have targeted individual, readily defined low-value services and shown modest reductions.^7,8,9,10^ A more holistic approach designed to improve testing decisions in general might capture a wider array of potentially low-value tests^11,12,13^ and cascades of downstream services of uncertain value that can follow from any test,^4,5,6,14^ along with potential broader benefits.

One approach to improve medical testing decisions is to encourage patients and clinicians to have routine shared decision-making conversations about any medical test under consideration. Although shared decision-making has largely been studied—and linked to better outcomes—for major decisions such as orthopedic surgery,^15^ some have argued for normalizing everyday shared decision-making for intermediate-stakes decisions when feasible.^16^ Priming short, meaningful conversations about even seemingly routine tests could address proposed drivers of overuse, including clinician norms,^17^ patient misperceptions and knowledge gaps,^18^ and inadequate patient-clinician communication.^19,20^ Such conversations could also contribute to patient-centered care by improving patients’ medical test literacy and allowing nuanced consideration of patient values and preferences in testing decisions.^16,21,22^ Yet, to our knowledge, no studies have implemented or tested an intervention that prepares clinicians and patients to understand and discuss medical testing decisions in general and during routine care.

We conducted a randomized clinical trial of a scalable, patient and physician-targeted intervention to promote high-quality conversations about the role and consequences of medical tests across 14 primary care clinics at an academic medical center. This just-in-time peer comparison and educational intervention was previously developed through user-centered design including multiple rounds of engagement with patients and clinicians.^18^ We focused on annual primary care visits as they are common and often involve low-value tests with potential to cascade.^11,14^ Our approach was further informed by studies showing that low-value care reduction interventions have greater efficacy when they are multicomponent and target clinicians as well as patients^23^; efficacy of physician peer comparison^24,25,26,27,28,29^; physician preference for point-of-care, electronic health record (EHR)–integrated reference materials^6,23^; and evidence that brief, just-in-time interventions improve outcomes.^15,30,31,32,33^ We hypothesized that this multimodal intervention would improve the quality of medical test conversations. As such, our primary outcome was a validated shared decision-making measure that captures discussion of benefits, harms, and alternatives. Secondarily, we examined whether the intervention would improve medical test knowledge and conversation satisfaction.

## Methods

### Study Design and Setting

We conducted a pragmatic, parallel group, matched-pair cluster randomized clinical trial with 2 groups (1:1 allocation ratio) across 14 primary care practices affiliated with Brigham and Women’s Hospital in Massachusetts. Details are summarized below and available in the trial protocol and statistical analysis plan in Supplement 1. We obtained informed consent from physicians via email and from patients verbally. The study was approved by Mass General Brigham and RAND institutional review boards and followed Consolidated Standards of Reporting Trials (CONSORT) reporting guidelines for randomized clinical trials.

### Physician Enrollment and Randomization

Our initial sample included Brigham and Women’s Hospital primary care physicians in active practice (ie, ≥50 visits conducted January 1, 2019, to January 1, 2021). For each physician, we extracted EHR data from this period to quantify rates of ordering 10 potentially low-value routine tests^34^ (complete blood count with or without differential, basic metabolic panel, liver function test, complete metabolic panel, urinalysis, thyroid stimulating hormone, vitamin D, electrocardiogram, and chest radiograph without relevant indication on problem list) during in-person annual visits that were not cobilled as problem-based visits. We ranked physicians by composite low-value testing rate and sequentially recruited physicians by decreasing rate among those with testing rates above the 25th percentile (best performers) of test ordering.

Physicians enrolled via email. For enrolled physicians, a research assistant used block matched-pair randomization and an Excel random number generator to randomize adjacently ranked physicians within gender groups to the intervention or control group. Upon study completion, all intervention group physicians were invited to participate in exit interviews.

### Patient Enrollment

Patients of enrolled physicians were eligible if they were aged 18 years or older, English-speaking, had email access, and had not opted out of research invitations. For each physician, we recruited at least 10 patients with scheduled in-person annual visits (study visits). We recruited patients for each matched physician pair sequentially and on a rolling basis to account for temporal trends. Patients were recruited via personalized portal-based letters that included links to the prestudy survey, with telephone follow-up for initial nonresponders. Among intervention group patients who completed the study, we used maximum variation sampling (according to age, gender, race and ethnicity, knowledge, preferences, and conversation content to capture a diversity of perspectives) to invite at least 1 patient per physician for exit interviews.

### Interventions

We administered interventions developed through user-centered design.^18^ One week before their first study visit, each intervention group physician received an email informing them they ordered more low-value tests than their peers (among physicians above the 50th percentile of low-value testing) or top-performing peers (among physicians in the 25th to 50th percentile of low-value testing), designed to motivate reconsideration of their testing approaches.^18^ The email included recommendations and both links and attachments of references on medical test interpretation and incidental findings that were also EHR-embedded and were designed to facilitate discussion with patients (eMethods 1, eMethods2, eAppendix 1, and eAppendix 2 in Supplement 2). In weeks with study visits, physicians were informed by email about the patient(s) scheduled that week. Control group physicians received emails with visit preparation tips (eMethods 3 in Supplement 2).

One to two days before their study visit, each intervention group patient received an email and text message with a link to the study website (eMethods 4 in Supplement 2). The website was titled “Medical Tests: The Basics” and included a video, quiz, and downloadable handout.^35^ Control group patients received email and text messages with visit preparation tips. Messages were sent using Research Electronic Data Capture (REDCap) software version 10.6.28 (Mass General Brigham).

### Data Collection

Using literature review and cognitive testing, we developed physician and patient prestudy and poststudy surveys (eMethods 5 in Supplement 2). Physicians received prestudy surveys before their first study visit and poststudy surveys after their patients had completed the study. Patients received prestudy surveys upon enrollment and poststudy surveys the day after their study visit. We used REDcap to administer surveys. Partway through the study, with IRB approval, we included an identifier in the weblink sent to the intervention group patients that allowed us to count website views.

We conducted semistructured physician and patient interviews about views on medical testing, experience with the intervention, knowledge gained, and study visit impressions (eMethods 6 in Supplement 2). For a post hoc exploratory analysis, we used medical record review to extract orders for each of the 10 aforementioned potentially low-value routine tests during study visits among intervention group and control group patients who completed the poststudy surveys.

### Measures

The primary outcome was the validated Shared Decision-Making Process survey (SDMP) score (0-4), which measures the extent to which patients are involved in a decision-making process.^22,36,37^ Secondary outcomes were the patient knowledge score (0-4), presence of test conversation, satisfaction with test conversation, presence of discussion of next steps, and whether the physician explained tests in a way that was easy to understand (all binary).

Exploratory patient outcomes included patient survey responses on which tests were discussed, who raised the idea of tests, and importance of various factors in testing decisions. These outcomes were captured using patient poststudy surveys. We also created a composite measure of potentially low-value routine tests ordered during study visits. Among intervention group patients, we calculated a lower-bound estimate of the proportion who viewed the website. Exploratory physician outcomes (physician poststudy survey) included importance of various factors in testing decisions; consideration of patient out-of-pocket costs; discussion with patients about false positives, incidental findings, and cascades; and barriers to cascade conversations.

Physician characteristics included time since residency, gender, race and ethnicity, percentage of professional time in outpatient practice, and prior experience with cascades (prestudy survey). Patient characteristics included age, gender, race and ethnicity, education (prestudy survey), years with primary care physician (PCP) (poststudy survey), and primary insurance (EHR).

### Statistical Analysis

#### Quantitative Analysis

Our primary analysis was intent to treat with multiple imputation of missing outcomes.^38^ We targeted a sample of 200 patient visits (10 per physician) based on estimated 80% or higher power to detect a 0.5-SD difference in SDMP between groups^39^ assuming a 2-sided type I error rate of 5% and intracluster correlation coefficient of 0.05 for patients from the same physician. We summarized baseline characteristics using means with SDs for continuous variables and frequencies with percentages for categorical variables.

##### Patient Outcomes

To estimate mean differences in patient outcomes between groups, we used linear mixed-effect models clustered by matched physician pair and physician nested within matched pair for continuous or ordinal outcomes. For binary outcomes, we used generalized estimating equations with a logit link clustered by matched physician pair. We then adjusted models for patient age, gender, education level, and race and ethnicity (given potential association with primary outcome^37,40,41,42,43,44^).

##### Exploratory Analyses

We compared patient exploratory outcomes using the above statistical tests. For physician exploratory outcomes, we described prestudy and poststudy results in the intervention and control groups. To explore how the intervention’s effect on primary and secondary patient outcomes varied by relevant subgroups, we stratified analyses by physician gender, years with PCP, and patient health care preferences.

We used multiple imputation to impute outcomes and years with PCP (postsurvey variable) for patients who did not complete the poststudy survey. Specifically, we used predictive mean matching with a fully conditional specification (robust to nonnormality of outcomes). We used R statistical software (version 4.2.2) and considered 2-sided *P* values significant at <.05. Data were analyzed from December 2022 to September 2023.

#### Qualitative Analysis

We transcribed interviews and analyzed them using an inductive thematic approach; 3 authors (I.G., E.C., and R.R.) developed a code book, coded data, reviewed coded data for key categories and themes, and resolved differences by consensus. We described frequency of themes. We also performed content analysis of open-ended patient responses to the postsurvey item on testing discussions.

## Results

### Sample Characteristics

**Figure 1.**
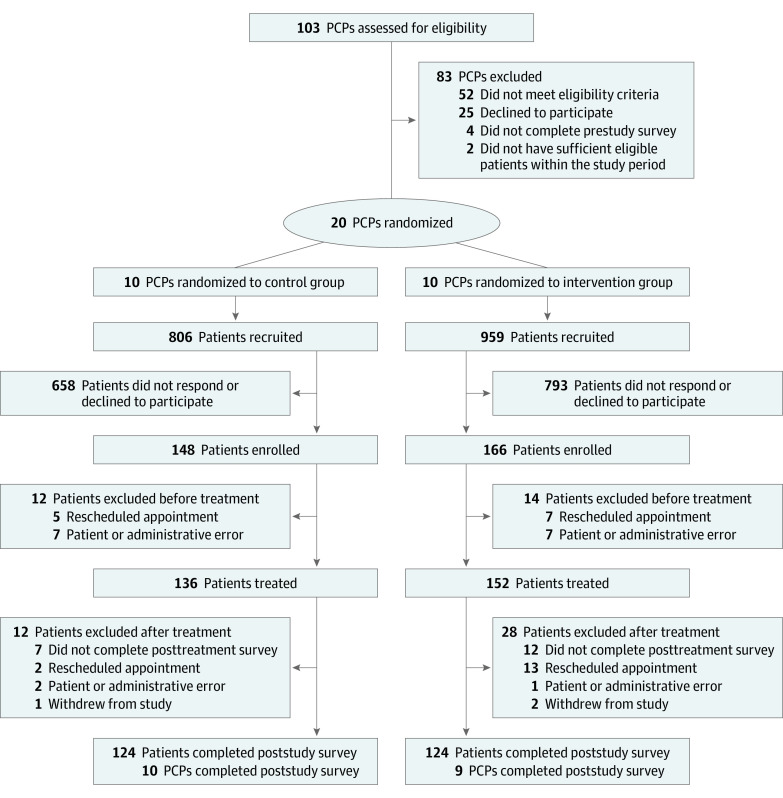
Trial Flow Diagram PCP indicates primary care physician.

Physicians were recruited May 24 through September 14, 2021. Among 51 invited physicians, 25 (49.0%) agreed to participate and 20 (39.2%) were included in the final sample (Figure 1). Of these 20 physicians, 13 (65.0%) were female and 13 (65.0%) were non-Hispanic White; mean (SD) time since residency was 19.7 (11.7) years; 14 (70.0%) spent at least 75% of their time in outpatient practice. Physicians randomized to intervention and control groups had similar baseline characteristics (Table 1). Nineteen physicians (95.0%) completed poststudy surveys.

**Table 1.** Physician and Patient Characteristics

Characteristic	Participants, No. (%)		
	All enrolled	Intervention group	Control group
**Physician characteristics**	**n = 20**	**n = 10**	**n = 10**
Gender			
Female	13 (65.0)	6 (60.0)	7 (70.0)
Male	7 (35.0)	4 (40.0)	3 (30.0)
Race and ethnicity			
Asian/Pacific Islander	4 (20.0)	2 (20.0)	2 (20.0)
Non-Hispanic White	13 (65.0)	7 (70.0)	6 (60.0)
Other^a^	3 (15.0)	1 (10.0)	2 (20.0)
Years since residency completion, mean (SD)	19.7 (11.7)	21.8 (14.5)	17.5 (8.2)
Time spent in outpatient practice, %			
25-74	6 (30.0)	5 (50.0)	1 (10.0)
>75	14 (70.0)	5 (50.0)	9 (90.0)
Experience with cascades			
Experienced once/y to a few times/y	11 (55.0)	5 (50.0)	6 (60.0)
Experienced once/mo to once/wk	9 (45.0)	5 (50.0)	4 (40.0)
**Patient characteristics**	**n = 314**	**n = 166**	**n = 148**
Age at time of visit, mean (SD)	50.2 (15.3)	50.6 (15.8)	49.8 (14.8)
Gender			
Female	210 (66.9)	107 (64.5)	103 (69.6)
Male	100 (31.8)	57 (34.3)	43 (29.1)
Other^b^	4 (1.3)	2 (1.2)	2 (1.4)
Race and ethnicity			
Asian/Pacific Islander	25 (8.0)	15 (9.0)	10 (6.8)
Hispanic	13 (4.1)	10 (6.0)	3 (2.0)
Non-Hispanic Black	10 (3.2)	6 (3.6)	4 (2.7)
Non-Hispanic White	246 (78.3)	126 (75.9)	120 (81.1)
Other^a^	20 (6.4)	9 (5.4)	11 (7.4)
Highest educational attainment			
No bachelor’s degree	39 (12.4)	12 (7.2)	27 (18.2)
Bachelor’s degree	118 (37.6)	68 (41.0)	50 (33.8)
Postgraduate degree	156 (49.7)	85 (51.2)	71 (48.0)
Primary Insurance			
Commercial	262 (83.4)	142 (85.5)	120 (81.1)
Medicaid	19 (6.1)	8 (4.8)	11 (7.4)
Medicare	26 (8.3)	14 (8.4)	12 (8.1)
Other^c^	7 (2.2)	2 (1.2)	5 (3.4)
Less than 3 y with current PCP^d^	138 (43.8)	55 (17.5)	83 (26.3)
Prefers to wait and see (vs take action on medical decisions)	191 (60.8)	103 (62)	88 (59.5)
Prefers patient makes final medical decision (vs physician/both)	133 (42.4)	72 (43.4)	61 (41.2)
How often does someone help read health materials?			
All or some of the time	49 (15.6)	25 (15.1)	24 (16.2)
A little of the time	78 (24.8)	42 (25.3)	36 (24.3)
None of the time	180 (57.3)	95 (57.2)	85 (57.4)
Not applicable	7 (2.2)	4 (2.4)	3 (2.0)

Abbreviation: PCP, primary care physician.

^a^ Other includes non-Hispanic of mixed racial background, some other race, and prefer not to answer.

^b^ Other includes gender variant or nonconforming and prefer not to answer.

^c^ Other includes self-insured, worker’s compensation, and UniCare Group Insurance Commission.

^d^ Years with PCP includes multiply imputed values for 74 patients.

Among 1765 recruited patients, 314 (17.8%) were enrolled and included in the final sample; 248 (79.0% of enrollees) completed poststudy surveys. Baseline characteristics were similar in intervention and control groups among all patients enrolled and among patients who enrolled and completed poststudy surveys (Table 1 and eTable 1 in Supplement 2). The mean (SD) age at the time of the physician visit was 50.2 (15.3), 210 patients (66.9%) were female, and 246 patients (78.3%) were non-Hispanic White.

### Outcomes

**Figure 2.**
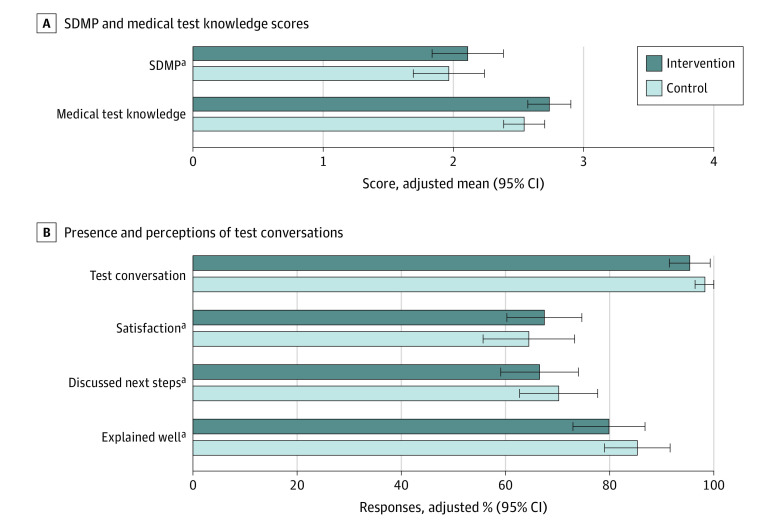
Effect of Peer Comparison and Educational Intervention on Medical Test Conversations A, adjusted means for Shared Decision-Making Process survey (SDMP) (0-4, higher indicates more shared decision-making) and medical test knowledge (0-4, higher indicates greater knowledge) scores for intervention and control groups with 95% CIs. B, adjusted percentages of “yes” responses for intervention and control groups with 95% CIs. All models included matched pair clustering and multiple imputation of missing values. ^a^Only asked if patient indicated presence of a test conversation.

Intervention group patients had slightly higher adjusted SDMP scores (2.11 vs 1.97 out of 4; difference, 0.14; 95% CI, −0.25 to 0.54) and knowledge scores (2.74 vs 2.54 out of 4; difference, 0.19; 95% CI, −0.05 to 0.43); these differences did not reach statistical significance (Figure 2 and eTable 2 in Supplement 2). Among the SDMP components, most patients reported discussing reasons to have tests; few reported discussing reasons not to have tests or alternatives to tests (eTable 3 in Supplement 2).

Compared with control group patients, intervention group patients were similarly likely to report any test conversation (95.4% vs 98.3%; difference, −2.9 percentage points; 95% CI, −7.0 to 1.2 percentage points). They were also similarly likely to report satisfaction with the conversation (70.7% vs 65.6%; difference, 5.1 percentage points; 95% CI, −6.5 to 16.7 percentage points), to report a discussion of next steps (69.8% vs 71.4%; difference, −1.7 percentage points; 95% CI, −12.8 to 9.5 percentage points), and to state their physician explained tests in a way that was easy to understand (83.7% vs 86.8%; difference, −3.1 percentage points; 95% CI, −12.7 to 6.6 percentage points).

Patients most commonly noted discussing blood tests (eTable 4 in Supplement 2). Most said they and their physicians both raised the idea of testing. Patients ranked “rule out a health problem” as most important in testing decisions, followed by “learn something about body/health.” In post hoc exploratory analysis of test ordering rates during intervention group versus control group study visits, we found no significant difference (mean [SD] 1.26 [2.19] tests per study visit in the intervention group; 1.34 [2.09] in the control group; difference, −0.09; 95% CI, −0.52 to 0.35). Eighty-three of 105 intervention group patients with trackable weblinks (79.0%) viewed the website.

In prestudy and poststudy surveys, physicians ranked patient out-of-pocket costs as most important in testing decisions, followed by desire to be thorough and fear of missing something (eTable 5 in Supplement 2). Most physicians reported that they considered patients’ out-of-pocket costs always or most of the time, but few reported talking to patients about the potential for incidental findings or cascades always or most of the time. Commonly reported barriers to discussing cascades were insufficient time and concern about confusing patients. In stratified analyses (eTable 6 and eTable 7 in Supplement 2), there were no differences by physician gender, years with PCP, or patient health care preferences.

### Qualitative Analysis

Interviewed intervention group physicians (3 physicians) and patients (16 patients) had similar characteristics to the full group (eTable 8 in Supplement 2). When patient participants were asked to describe reactions to study materials, many (defined as 4-8 patients) recalled not paying close attention (Table 2). Some (defined as 2-3) attributed this to high volumes of other previsit communications; most said the general, accessible language made them believe they already knew the content and were not the target audience. Many patients remembered content on incidental findings and false positives, which 1 described as “scary.” Some believed the materials were useful reminders or confirmations of their knowledge.

**Table 2.** Key Themes and Quotes From Patient and Physician Interviews

Themes	Quotes^a^
Patient interviews	
Patient reactions to educational materials	
Level of engagement	“Just all standard stuff that you’d expect.” (273) “It was kind of, it was… rudimentary, and it was, you know, it was nothing that surprised me. So I think it’s one of those things where in a brain that’s overloaded, it probably, not too much stuck.” (378)
Perceived utility	“I think it was definitely useful you know… I did have a few things I wanted to discuss with my doctor… maybe annual blood work isn’t that necessary, especially at my age… some of the challenge with the annual bloodwork, it’s kind of one of those things where you’re just desensitized to [it] at this point from [childhood], that it’s just expected.” (216) “I mean, I think it’s always good to just kind of refresh of like why you’re having an annual exam, are things you could ask or just like reminders of things that you might have forgot, that you have come up over the year.” (239)
Interpretation of purpose	“I was already sold on the idea of adhering to my doctor’s advice and I didn’t have any particular fear or apprehension about it.” (63) “It was [trying to make] a patient to be comfortable in advocating for themselves in, you know, in a situation where maybe they didn’t feel comfortable doing a test, that’s the way I read it… it was sort of encouraging you maybe to take more charge of your own health care decisions.” (79) “I think it encourages people to, you know, as they go in to see their doctor, you know, write, and I would, you know, I’d always encourage people to write down stuff. And bring a list in.” (215)
Patient views on testing and health care	
Mostly positive views toward medical testing	“The more the better. Never been opposed to testing. I believe in good health, I believe in preventative care, and I also like to know what’s going on inside of me so… I’m open to pretty much all testing.” (215) “I have an objectively positive feeling that medical tests are helpful and useful in keeping people healthy. But obviously a personal distaste for discomfort and inconvenience.” (63) Reads aloud from study materials: “‘Medical tests have possible benefits and possible downsides.’ Sure, I mean downsides gives you a little pause of is there danger here? Do I need to worry about it? But, you know, not really.” (63) “I would have liked to have a more thorough check-in, so let’s see also your legs, let’s have an electrode in your heart, just to make, you know, just to see the whole thing and see how it’s doing.” (204)
Mostly negative or mixed views of medical testing	“I’ve also been a very intimate witness of what’s going on with my mother in her old age, and I feel like it’s ridiculous sometimes what they do in terms of giving her tests. I mean it’s so redundant, and so unnecessary, and so costly.” (79) “I used to work in an area where we ordered a lot of testing and some patients would ask for every test under the sun… it’s definitely not always the best option, and, you know, for [cost’s sake] too I guess.” (158) “I don’t want to get, you know, tests done routinely that might be, yeah, OK, and then we’re down the rabbit hole looking for something that’s wrong when there’s no symptom… I’m going to be very, very thoughtful about the tests that are ordered on me.” (185) “Going through a battery of tests you know is um, inconvenient and takes time, and if you’re a busy person, you know I don’t want to do that unless I’m really sure it’s warranted. I’m also aware of the issues related to false positives or excessive treatment.” (233) “Part of this is sort of like not particularly wanting to live constantly being tested for cancer... I mean AHH! It’s just no way to live… as a patient personally, I find it anxiety producing to be constantly told, ‘We have to test you for cancer’ because my understanding is if you look hard enough, you’re gonna find something, and I’m not sure why I should go through that sort of morbid curiosity.” (236) “I tend to wait, you know, wait before I, to see whether, you know, tests are necessary… I know my own body and… I’m conscious about costs, I’m conscious about my time and, you know, just because there’s access to medicine doesn’t mean I have to use it.” (273) “Even blood tests, you know, sticking a needle in you. There’s, anything that, that, uh that enters your body, there is a downside.” (273)
High trust in doctor’s advice on medical test decisions	“I mean I’m always happy to do anything routine….I trust my doctor, if it’s something that he maybe recommends it’s something I should do.” (158) “I feel like he’ll make a recommendation but also ask what I feel like and I’ll, I’m happy to provide my opinion, but I also kind of trust him a bit more than my opinion.” (158) “I always go with the flow, whatever they say to me is good, and I don’t question them. I’m a firm believer in doctors. So whatever they tell me, I always say, okay, okay.” (218) “I know from previous experience that when you have an annual physical that’s always part of it is they want to monitor your, you know, the counts on cholesterol and things like that… [and] there were some reasons so to follow up on the medicines I’m taking… also it had been probably a year… since I had lab work done.” (79) “If I had to get a second opinion on something that was very serious, you know I might… But normally I’m not worried about my primary care doctor and her knowledge of my own treatment and my own history.” (239)
Patient perceptions of whether or how study materials influenced study visit	
No perceived influence	“You’re asking if there was any difference in sort of the experience I had with her from… since last year… I think there was a certain level of comfort, because we knew each other. But no, other than that, no.” (185) “Um nothing different, um, kind of just like went through and like checked in on how I was doing with all the things that she was following up on. And then did an exam and, that was kind of yeah. It was nothing really unusual that happened.” (156)
Perceived influence	“And I actually had a better conversation with my doctor that day, ‘cause I kept on remembering that study… It was an eye opener… I am more able to understand the test of time.” (218)
Physician interviews	
Physician views on overtesting	
Role of patient preferences in overtesting	“I didn’t start off [in] medicine always giving labs, but my patients are a little bit like, I want this, I want this. So I’ve like, felt defeated and I’m like, I’m not gonna fight these battles ‘cause it just doesn’t seem like they’re worth the fight.” (4) “In general, I, you know, it’s interesting, I have patients that come in that often will say, you know, ‘I have great insurance, I want you to order whatever, you know, order everything.’” (8) “I will often put to the patient the possibility of not doing bloods on a given visit and deferring it. What I had found repeatedly, I would say the vast majority, when I posed that option, people say I want it.” (9)
Role of physician preferences and culture of medicine in overtesting	“I don’t wanna miss anything. I’m protective of my patients and you know, in those instances where you feel like you didn’t do something that you could have done and things didn’t turn out well, I mean that that… that kind of scenario definitely plays out in my mind.” (9) “It’s working a little bit against some sort of ingrained habitual, you know, thing where you’re like, okay, annual physical, CBC, CMP, like the whole 9 yards.” (8)
Role of visit time constraints in overtesting	“I give in because I just don’t have the time for [talking patients out of getting low-value tests], you know?” (4) “You have maybe 20 minutes or 18 minutes left to do your physical. And they’re like, oh, I brought my list with me… everything is so cramped.” (8)
Physician perceptions of how study materials influenced their practice	“I usually [discuss what labs are or aren’t needed] anyways, but… rather than defaulting to… what I felt like the patient needed, I just tried to take the extra minute to explain to them what labs I felt like was only essential or not essential, or explain to them, you know what, you’re 40. You’re young and healthy, you’re doing all the right things, so you actually don’t need labs every year. You can afford to have labs every 3 to 5 years or whatever the situation is.” (4) “I am more mindful of [applying lessons from the study references] when I am ordering because I recognize the amount of downstream work I end up stuck with when I have all these abnormal labs.” (4) “I think that I think it’s definitely, it’s definitely changed… I think you can recognize quickly who’s willing to hear the information, and will have a shift in thinking, so it’s worth having that conversation.” (8) “In situations where I felt like the testing was not strongly needed, kind of elective, you know, in those where I could pose to the patient the option not doing it, I think I probably, as a result of being in the study, maybe did that a little more often perhaps.” (9) “I’m not gonna spend a lot of time sort of saying, well you know, this [laboratory test] could turn out abnormal and this may cause you a lot of inconvenience and worry and, you know, I just feel like I have higher value targets. So I, I guess I don’t prioritize that. I think a lot harder about other, you know, much more costly, invasive or potentially impactful tests.” (9)

^a^ Parenthetical numbers are unique participant identifiers.

When asked their views on testing, most patients expressed willingness to get tests that were routine, recommended by their physicians, or perceived as not burdensome. Some disagreed with study material content about testing downsides but most expressed prior understanding of these downsides, influenced by their own or their family’s or friends’ experiences with tests, health care training or, in 1 case, news about false positives triggering care cascades.

Most patients described receiving test(s) during their study visit. Many patients said their physicians made the decision with little or no discussion; all patients said they would have felt comfortable asking questions if they had any. Many stated they trusted their physicians to recommend necessary tests. Most also said their physicians had discussed these tests with them previously, they had prior knowledge of tests, or they expected some routine tests during annual examinations.

When physician participants were asked about reactions to the peer comparison email and materials, all physicians recalled having ordered unnecessary tests by patient request. Two noted a habit of overtesting that they wanted to break; the third shared a fear of missing something. When asked about impact of the materials, all described being at least somewhat more likely to discuss possible downsides of testing with patients after the study. However, they noted barriers to convincing patients to avoid certain tests (eg, “I give in because I just don’t have the time”). Given these constraints, 2 focused on discouraging low-value tests with more cascade potential, such as imaging for uncomplicated low back pain and prostate cancer screening. One physician suggested formal training to develop elevator pitches about test downsides.

In our analysis of 68 open-ended patient survey responses, many justified a lack of discussion, most commonly by stating their tests were routine (eTable 9 in Supplement 2). Several reported discussing that no tests were needed; 1 reported their physician ordered tests that were not discussed. No negative consequences of the intervention emerged.

## Discussion

In this initial randomized trial of a physician-facing and patient-facing peer comparison and educational intervention, we did not observe statistically or clinically significant^22,45^ improvement in shared decision-making conversations about testing during annual visits. Nearly all participants across groups reported discussing 1 or more tests during these visits, indicating opportunities to improve conversations about potentially low-value tests. Qualitative analyses revealed addressable barriers. For example, while some interviewed physicians said peer comparison and reference materials helped them shift their approach to low-value testing, all perceived insufficient time to discuss potential downsides of tests. Many patients did not feel the educational materials applied to them, did not observe meaningful changes in their physicians’ approaches, and trusted their physicians’ advice even when inconsistent with materials.

Our educational intervention was designed to improve patient-clinician conversations by bolstering general medical test literacy, in contrast to prior studies evaluating decision aids for specific decisions such as knee surgery^46,47,48^ and behavioral nudges to reduce specific services such as inappropriate antibiotic or antipsychotic medications.^24,25,28^ We chose this approach to accommodate simple scale-up and influence a range of testing decisions (we also included specific examples in materials in response to user feedback). However, lack of specificity to a single test or clinical scenario may have diluted the intervention’s true impact, and/or its impact as measured by SDMP. For physicians, sharing the references may have been insufficient to overcome entrenched habits or lack of familiarity with discussing concepts such as test characteristics and cascades.^49^

Although competing demands are a real issue in packed primary care visits,^50,51,52^ physicians’ perceptions of insufficient visit time may instead reflect the time or effort required to change practice habits (eg, using scripted language provided in the references).^16^ Discussing tradeoffs of testing and counseling against potentially low-value tests is likely faster than perceived. As some interviewed physicians noted, such counseling may also decrease time spent discussing these tests at future visits, as well as the substantial time PCPs spend following up normal results and abnormal results that can cascade.^4,6,14,18,53^

Our results highlight challenges in intervening on trusting patient-physician relationships with established testing norms. Our study included PCPs with higher testing rates; patients choosing to stay with these physicians likely had concordant preferences. Interestingly, among patients who had known their PCP for less than 3 years, intervention and control group patients still had similar outcomes. We found patients trusted their physicians to offer only necessary tests. At the same time, physicians assumed their patients wanted more tests, though evidence suggests otherwise: patients wish their physicians talked to them about potential incidental findings before they occurred^20^ and report that their physicians de-implementing low-value screening tests would not harm patient-physician relationships and might even increase trust.^54^ What’s more, patients exposed to more low-value care do not rate their patient experiences more highly.^55^ These cycles of misunderstanding proved difficult to break.

Our results also reflect the engrained more-is-more culture in health care (which may be stronger among patients choosing to attend annual examinations),^19^ and the pervasive idea that routine testing is unequivocally beneficial and definitive (eg, to rule out a health problem) and therefore may not require a shared decision-making conversation. To this point, 89% of intervention group patients and 97% of control group patients incorrectly answered that routine yearly screening tests are useful in most cases. Although we tested materials with users, further refinement of this messaging is critical to reach diverse audiences.

Our results suggest that test conversations remain an important target for intervention. In future iterations, the physician intervention may require additional components such as clinic-wide implementation, support from clinic directors or local clinical champions,^56^ practical learning sessions, or peer coaching.^57^ In surveys, most physicians said they frequently considered patient out-of-pocket costs in clinical decisions, yet meaningful conversations on this topic may nevertheless be limited by lack of information—when available, patient cost calculators might fill this gap.^58,59^ Future patient educational materials might focus on specific prototypic tests to build medical test literacy. These materials could include testing data from one’s PCP or otherwise leverage trusting relationships, though customization may reduce scalability. Future research should further examine the relationship between test conversations and overuse, a potential role for incentives, and the extent to which clinician readiness for change^60^ may predict intervention efficacy.

### Strengths and Limitations

This randomized clinical trial had several strengths, including use of a scalable intervention developed with user-centered design that was targeted to physicians and patients and evaluated using mixed methods. It also had limitations. For example, we could not blind participants to study group. Participants included English-speaking patients with email access in an academic medical setting and were largely well-educated and White, limiting generalizability beyond this population. Only 18% of recruited patients were enrolled, which may have resulted in a patient sample that was more engaged than the general population. Results may be limited by recall bias. We were not able to capture how often physicians accessed reference materials since we supplied them via multiple modalities (including email attachments), though qualitative results suggest at least some did review them carefully. Additionally, the intervention may have caused some physicians to not offer low-value tests in the first place, which would preclude our primary outcome of conversation quality (in support of this, fewer intervention group patients reported discussing a test with their physician and fewer potentially low-value tests were ordered during their study visits, though these differences were not statistically significant).

## Conclusions

In this initial randomized trial of a physician-facing and patient-facing peer comparison and educational intervention, we did not find significant improvement in shared decision-making conversations about medical testing during annual visits. Testing was common during annual examinations and there was evidence of cycles of misunderstanding in which patients and physicians assumed the other party wanted testing, with many patients not fully appreciating potential downsides. To break these cycles, improve conversations around medical tests, and reduce overuse and downstream cascades, future efforts should involve robust, targeted interventions that focus on mitigating physician adoption barriers and further leveraging patient-clinician relationships.

## Notes

Supplement 1.
Trial Protocol

Supplement 2. **eMethods 1. **Physician Peer Comparison Sample Email **eMethods 2. **Reference Materials for Intervention Group Physicians **eAppendix 1. **Physician Reference Materials on Interpretation of Medical Tests **eAppendix 2. **Physician Reference Materials on Incidental Findings **eMethods 3. **Physician Control Group Email **eMethods 4. **Reference Materials for Intervention Group Patients **eMethods 5. **Survey Instruments **eMethods 6. **Exit Interview Guides **eTable 1. **Baseline Patient Characteristics Stratified by Final Survey Completion Status **eTable 2. **Comparison of Patient-Reported Test Conversation Measures by Treatment Group **eTable 3. **Patient Responses to Individual Components of SDMP and Medical Test Knowledge Scores by Treatment Group **eTable 4. **Comparison of Patient-Reported Visit Descriptions and Factors Important to Testing Decisions by Treatment Group **eTable 5. **Summary of Physician Test Ordering Preferences and Behaviors, Prestudy and Poststudy, by Treatment Group **eTable 6. **Patient Analyses Stratified by Physician Gender and Patient Years With PCP **eTable 7. **Patient Analyses Stratified by Patient Preferences About Medical Decision-Making **eTable 8. **Baseline Characteristics of Interviewed Physicians and Patients **eTable 9. **Summary of Qualitative Patient Descriptions of Test Conversation During Study Visit
eReferences

Supplement 3.
Data Sharing Statement
