# Supplementary material for: Effect of a Peer Comparison and Educational Intervention on Medical Test Conversation Quality: A Randomized Clinical Trial
Source: JAMA Netw Open. 2023 Nov 9;6(11):e2342464. doi: 10.1001/jamanetworkopen.2023.42464 (PMC10636635; doi:10.1001/jamanetworkopen.2023.42464)
Supplement: Supplement 1. — Trial Protocol [file jamanetwopen-e2342464-s001.pdf]

Supplementary Material 1: Trial protocol and statistical analysis plan

Table of Contents

Original Protocol 2
Final Protocol 15
Statistical Analysis Plan 29

Original Protocol

PRINCIPAL/OVERALL INVESTIGATOR

Ishani Ganguli MD MPH

PROTOCOL TITLE

CASCADES Trial: A consumer-focused approach to initiate shared decision-making on care cascades after common medical tests

FUNDING

Robert Wood Johnson Foundation

VERSION DATE

3/24/2021

SPECIFIC AIMS

Concisely state the objectives of the study and the hypothesis being tested.

Aims:

1. Develop a simple, scalable intervention to prompt conversations about the downstream consequences of potentially discretionary medical tests in the primary care setting.
2. Implement this text-based intervention in the primary care setting.
3. Rigorously evaluate the impact of this intervention using a randomized controlled trial study of 200 patients by their primary care physician.

Hypotheses:

1. The intervention is feasible to implement in primary care practices with no major recruitment barriers as defined by recruiting 20 physicians from clinics affiliated with one academic medical center, and 200 patients of all sexes and a range of ages, ethnicities, income levels (as correlated with zip code), and education levels.
2. Intervention arm patients are more likely to have higher quality conversations about medical testing decisions (primary outcome).

BACKGROUND AND SIGNIFICANCE

Provide a brief paragraph summarizing prior experience important for understanding the proposed study and procedures.

Medical tests can have sizable downstream consequences including further tests, treatments, office visits, and even hospitalizations. Known as *care cascades*, these downstream services have significant direct and indirect cost implications for patients. In some cases, downstream services are medically appropriate, such as when an initial test is medically indicated and has a high degree of accuracy. However, many tests are performed even when they are not medically indicated (e.g., at a patient's request), and may have high rates of false positives or of incidental findings (i.e., unrelated to the purpose of the test). Notably, the following tests are commonly ordered, may be overused, and have a high risk of false positives and incidental findings (and therefore care cascades): imaging tests, electrocardiograms, and blood tests including blood count, electrolyte, kidney function, and liver function tests. Some estimates suggest that up to 52% of radiology and laboratory tests produce incidental findings, and that rate may increase with advances in technology.

Studies of specific cascades using national administrative claims data also suggest that these cascades can be costly – in one example by Ganguli et al, cascades following pre-operative electrocardiograms for cataract surgery cost ten times the initial electrocardiograms (ECGs). A recent national study of US internists conducted by Ganguli et al. also found that almost all responding physicians had experienced cascades after incidental findings that did not lead to clinically meaningful outcomes yet caused physical, psychological, or other harms to patients or the physicians themselves. Although some of these cascades may eventually reveal clinically important findings, more often they find nothing significant. This is especially true when the initial test is discretionary or even known to be of low-value, as in the example of pre-operative ECGs for cataract surgery. It is likely that few patients are aware of the potential for cascades or risk of false positives when making the decision with their clinicians to proceed with medical tests.

Despite the prevalence and burden of care cascades, little effort has been devoted to addressing them. Consideration of care cascades have not been incorporated into best practices for shared decision-making and we are not

aware of any interventions that have been developed or evaluated to educate patients or clinicians on the financial or psychological risk of care cascades.

RESEARCH DESIGN AND METHODS

Briefly describe study design and anticipated enrollment, i.e., number of subjects to be enrolled by researchers study-wide and by Partners researchers. Provide a brief summary of the eligibility criteria (for example, age range, gender, medical condition). Include any local site restrictions, for example, "Enrollment at Partners will be limited to adults although the sponsor's protocol is open to both children and adults."

Design: During Phase I, we will implement a user-centered design process to develop the educational content and implementation strategy for the intervention. Specifically, we will conduct focus groups with patients and clinicians in which we iteratively solicit feedback on the language and dissemination of text/email-based messages and interactive content for patients, on the tip sheet for physicians, and on survey items for both.

In Phase II, we will randomize 20 primary care physicians to intervention and control arms, recruit 10 patients per physician with upcoming wellness visits, and implement our intervention. We will collect pre- and post-intervention surveys of patients and physicians, as well as post-study interviews of patients and physicians. In Phase III, we will use mixed methods analysis to study the data collected in Phase II.

Enrollment: For Phase I, we will recruit up to 20 patients for the patient advisory committee and interviews and up to 20 practicing BWH primary care physicians for interviews.

For phase II, we will recruit 20 BWH primary care physicians who actively see patients and meet our inclusion criteria (based on patient-deidentified EDW data on test ordering rates during annual physicals). We will then recruit 10 patients for each of these PCPs. All patients of the selected PCPs who are at least 18 years old, speak English, and have access to Email are eligible to participate.

Briefly describe study procedures. Include any local site restrictions, for example, "Subjects enrolled at Partners will not participate in the pharmacokinetic portion of the study." Describe study endpoints.

In phase I, we will develop educational content for patients and a tip sheet for providers. To this end, we will work with the BWH Center for Patients and Families to select and convene a 5-10 member patient advisory council. We will host two focus groups with this council, aided by discussion guides, to first identify key issues and then to suggest refinements to our intervention.

We will also recruit both patients and physicians for in-depth interviews. We will recruit up to 20 primary care physicians through the Patient-Based Research Network (PBRN) and suggestions from clinic medical directors. We will recruit up to 20 patients through the above Patient Advisory Council and the Rally platform. We will conduct up to 20 in-depth interviews each with these physicians and patients to understand their experiences with cascades and to cognitively test survey items.

Finally, we will review patient educational materials and the clinician tip sheet with clinic medical directors for their approval.

In phase II, we will work with clinic leads in the BWH PBRN to recruit 20 physicians who have not previously been involved. We will select these physicians from among those who fall in the top 50th percentile of test ordering rates during annual physicals. We will use matched pair randomization to randomize these PCPs to the control or intervention arms. For each physician, we will identify 10 of their patients who are scheduled for a physical in the next 6 months and meet our inclusion criteria (based on key demographics from the electronic health record (EHR)). We will recruit them using use mailed letters and patient portal messages.

Intervention arm: We will send emails to physicians with feedback on how they compare to their peers in aggregate on test ordering during annual physicals, along with links to the physician-facing materials. We will not share identifiable data with a given physician on their peer physicians. One to two days before their visit, we will send patients educational materials via text, email, or both depending on their preference.

Control arm: One to two days before their visit, we will send patients general information on visit preparation.

NOTE: We acknowledge that during the covid19 pandemic, clinical operations have changed drastically to protect both patients and health care workers. For instance, routine wellness visits have been cancelled or converted to virtual. Given our commitment to protect the safety and welfare of our study subjects and staff, and to avoid disrupting clinic operations during this challenging time, we plan to conduct phase I of our study virtually and to begin the intervention phase only once it is deemed safe and feasible to do so.

> For studies involving treatment or diagnosis, provide information about standard of care at Partners (e.g., BWH, MGH) and indicate how the study procedures differ from standard care. Provide information on available alternative treatments, procedures, or methods of diagnosis.

Treatment and diagnosis are not part of this protocol.

> Describe how risks to subjects are minimized, for example, by using procedures which are consistent with sound research design and which do not unnecessarily expose subjects to risk or by using procedures already being performed on the subject for diagnostic or treatment purposes.

There are no known risks to participating in this study. Patient and clinician subjects may voluntarily participate and withdraw at any time.

There is a theoretical risk of breach of data confidentiality and that PHI could become known to unauthorized persons, but we will take all steps necessary to protect PHI (see below). To mitigate this risk, we will follow all compliance and data confidentiality procedures for research at Partners. Specifically, we will use procedures consistent with sound research design and which do not expose subjects to unnecessary risk. Data monitoring will be conducted regularly (see below). All data analyzed will be de-identified and reported in aggregate.

> Describe explicitly the methods for ensuring the safety of subjects. Provide objective criteria for removing a subject from the study, for example, objective criteria for worsening disease/lack of improvement and/or unacceptable adverse events. The inclusion of objective drop criteria is especially important in studies designed with placebo control groups.

This is a minimal risk study. There is no known physical or medical safety threat to patient or clinician subjects who participate. Furthermore, participation is voluntary and all subjects will be informed that they may opt-out any time (see below). Given the minimal risk to subjects, our data safety and monitoring procedures, and the relatively small sample size, we do not plan on having drop criteria.

FORESEEABLE RISKS AND DISCOMFORTS

> Provide a brief description of any foreseeable risks and discomforts to subjects. Include those related to drugs/devices/procedures being studied and/or administered/performed solely for research purposes. In addition, include psychosocial risks, and risks related to privacy and confidentiality. When applicable, describe risks to a developing fetus or nursing infant.

For patient and clinician subjects, there are no known risks to participating in our data collection activities. There is a potential risk of physicians feeling psychological discomfort in knowing their test ordering rates. We will mitigate this by offering an option if they have concerns to speak with clinic leaders about these concerns. The main risks are breach of confidentiality and privacy of information shared. Every effort will be made to maintain confidentiality and privacy of information shared and collected and subsequent data analysis (see below), and efforts will be made to minimize the duration of data collection activities.

EXPECTED BENEFITS

> Describe both the expected benefits to individual subjects participating in the research and the importance of the knowledge that may reasonably be expected to result from the study. Provide a brief, realistic summary of potential benefits to subjects, for example, "It is hoped that the treatment will result in a partial reduction in tumor size in at least 25% of the enrolled subjects." Indicate how the results of the study will benefit future patients with the disease/condition being studied and/or society, e.g., through increased knowledge of human physiology or behavior, improved safety, or technological advances.

Individual subjects may benefit by learning about (and, if appropriate, avoiding) possible downstream consequences of medical testing, and having their physicians become more informed about these consequences and how to discuss them. Future patients will benefit through improved knowledge of a possible intervention to facilitate patient-clinician conversations about cascades that may in turn help to reduce their negative effects.

EQUITABLE SELECTION OF SUBJECTS

The risks and benefits of the research must be fairly distributed among the populations that stand to benefit from it. No group of persons, for example, men, women, pregnant women, children, and minorities, should be categorically excluded from the research without a good scientific or ethical reason to do so. Please provide the basis for concluding that the study population is representative of the population that stands to potentially benefit from this research.

Our goal will be to enroll eligible patients whose characteristics are representative of the entire population of eligible patients. We will specifically target patients of all sexes and a range of ages, ethnicities, income levels and education levels.

When people who do not speak English are excluded from participation in the research, provide the scientific rationale for doing so. Individuals who do not speak English should not be denied participation in research simply because it is inconvenient to translate the consent form in different languages and to have an interpreter present.

We will approach English-speaking patients only. The scientific rationale for this limitation is because the text-based intervention will be available in English in this early stage of development. All clinician subjects caring for patients at BWH speak English.

For guidance, refer to the following Partners policy:

Obtaining and Documenting Informed Consent of Subjects who do not Speak English
https://partnershealthcare-public.sharepoint.com/ClinicalResearch/Non-English_Speaking_Subjects.1.10.pdf

RECRUITMENT PROCEDURES

Explain in detail the specific methodology that will be used to recruit subjects. Specifically address how, when, where and by whom subjects will be identified and approached about

participation. Include any specific recruitment methods used to enhance recruitment of women and minorities.

For phase I, we will recruit the PAC and physician focus groups as described above.

For phase II, we will work with clinic leads in the BWH PBRN to recruit 20 physicians who have not previously been involved with our study. We will select these physicians from among those who fall in the top 50th percentile of test ordering rates during annual physicals, starting from PCPs with the highest test ordering rates and proceeding in descending order. Once we have recruited 20 physicians, we will use matched pair randomization to randomize these PCPs to the control or intervention arms.

Eligible patient participants will then be identified by research assistants trained in the protection of human subjects based on upcoming appointment and demographics including age, race, sex, and zip code (to link to area-level census data on income and education) using “minimum necessary” information in the EHR. Once identified, the research assistant will send the patient both a letter through the patient portal and a mailed letter inviting them to participate on behalf of the BWH PI. In the letter we will include an email address and phone number that allows the patient to opt out. The letter will also include a link to REDCAP which will include an electronic consent form with information sheet, invitation to enroll, preference for communication (phone versus email), and the baseline survey.

Provide details of remuneration, when applicable. Even when subjects may derive medical benefit from participation, it is often the case that extra hospital visits, meals at the hospital, parking fees or other inconveniences will result in additional out-of-pocket expenses related to study participation. Investigators may wish to consider providing reimbursement for such expenses when funding is available

Patient subjects in both arms will be compensated $10 at the completion of the study. Physician subjects will be compensated $50 for the whole study.

For guidance, refer to the following Partners policies:

Recruitment of Research Subjects

https://partnershealthcare-public.sharepoint.com/ClinicalResearch/Recruitment_Of_Research_Subjects.pdf

Guidelines for Advertisements for Recruiting Subjects

https://partnershealthcare-public.sharepoint.com/ClinicalResearch/Guidelines_For_Advertisements.1.11.pdf

Remuneration for Research Subjects

https://partnershealthcare-public.sharepoint.com/ClinicalResearch/Remuneration_for_Research_Subjects.pdf

CONSENT PROCEDURES

Explain in detail how, when, where, and by whom consent is obtained, and the timing of consent (i.e., how long subjects will be given to consider participation). For most studies involving more than minimal risk and all studies involving investigational drugs/devices, a licensed physician investigator must obtain informed consent. When subjects are to be enrolled from among the investigators' own patients, describe how the potential for coercion will be avoided.

In phase I, eligible physician subjects will be informed about our research initiative via email and patients via RALLY. Prior to participation in interviews, we will confirm verbal consent. Specifically, these subjects will be informed that: 1) participation is voluntary–they may stop the interview at any time, and 2) data collected will be analyzed and reported in aggregate.

In phase II, potential patient subjects will be informed as part of the letter or patient portal message, and the REDCAP link that it will contain. This information will contain the purpose of the study, risks/benefits, methods of ensuring confidentiality, and voluntary nature of participation. Specifically, patient subjects will be informed that: 1) participation is voluntary–they may withdraw at any time; 2) a decision to not participate will in no way affect their care at BWH/BWFH; 3) information shared will remain confidential and used only for research purposes; and 4) data collected will be analyzed and reported in aggregate. We will not request verbal consent as this will be prohibitively burdensome for this minimal risk study and because this study does not share any personal health information with the patients.

NOTE: When subjects are unable to give consent due to age (minors) or impaired decision-making capacity, complete the forms for Research Involving Children as Subjects of Research

and/or Research Involving Individuals with Impaired Decision-making Capacity, available on the New Submissions page on the PHRC website:

https://partnershealthcare.sharepoint.com/sites/phrmApply/aieipa/irb

For guidance, refer to the following Partners policy:

Informed Consent of Research Subjects:
https://partnershealthcare-public.sharepoint.com/ClinicalResearch/Informed_Consent_of_Research_Subjects.pdf

DATA AND SAFETY MONITORING

Describe the plan for monitoring the data to ensure the safety of subjects. The plan should include a brief description of (1) the safety and/or efficacy data that will be reviewed; (2) the planned frequency of review; and (3) who will be responsible for this review and for determining whether the research should be altered or stopped. Include a brief description of any stopping rules for the study, when appropriate. Depending upon the risk, size and complexity of the study, the investigator, an expert group, an independent Data and Safety Monitoring Board (DSMB) or others might be assigned primary responsibility for this monitoring activity.

NOTE: Regardless of data and safety monitoring plans by the sponsor or others, the principal investigator is ultimately responsible for protecting the rights, safety, and welfare of subjects under his/her care.

The principal investigator and research assistant/project coordinator will monitor data collection activities, maintain data integrity and quality control, protect the rights, safety, and welfare of study subjects, and adhere to standards set by the Partners IRB. All data will be stripped of PHI and electronic data will be kept on a secured shared file area (SFA) behind the Partners firewall with anti-virus software or Partners Research Computing approved cloud storage services. Data about consented patients will also be stored behind the RAND Corporation firewall in accordance with the terms of the consent. Any paper forms and/or audio recordings will be destroyed after being transcribed into electronic format. Monitoring will be performed on a monthly basis during the study period and will include a review of enrollment, data analysis, and breaches of confidentiality, and any adverse events that may occur. Any adverse events will be graded as to their

attribution to the intervention and reported according to IRB guidelines. The principal investigator will review any complaints reported from patient and physician subjects. These will be reported to the Partners IRB annually or immediately if the complaint is serious. Given the minimal risk of the study, we are not planning to use automatic stopping rules or a DSMB. We emphasize that this study does not involve any invasive procedures.

> Describe the plan to be followed by the Principal Investigator/study staff for review of adverse events experienced by subjects under his/her care, and when applicable, for review of sponsor safety reports and DSMB reports. Describe the plan for reporting adverse events to the sponsor and the Partners' IRB and, when applicable, for submitting sponsor safety reports and DSMB reports to the Partners' IRBs. When the investigator is also the sponsor of the IND/IDE, include the plan for reporting of adverse events to the FDA and, when applicable, to investigators at other sites.
>
> NOTE: In addition to the adverse event reporting requirements of the sponsor, the principal investigator must follow the Partners Human Research Committee guidelines for Adverse Event Reporting

Voluntary participation in data collection activities are not expected to cause adverse events to patient or physician subjects. However, if an adverse event occurs, the principal investigator will follow PHS HRC guidelines for adverse event reporting.

MONITORING AND QUALITY ASSURANCE

> Describe the plan to be followed by the principal investigator/study staff to monitor and assure the validity and integrity of the data and adherence to the IRB-approved protocol. Specify who will be responsible for monitoring, and the planned frequency of monitoring. For example, specify who will review the accuracy and completeness of case report form entries, source documents, and informed consent.
>
> NOTE: Regardless of monitoring plans by the sponsor or others, the principal investigator is ultimately responsible for ensuring that the study is conducted at his/her investigative site in accordance with the IRB-approved protocol, and applicable regulations and requirements of the IRB.

The principal investigator and research assistant/project coordinator will monitor and assure the validity and integrity of the data collection and adherence to the procedures outlined in this protocol. They will review the

study database on a monthly basis, ensuring data integrity, accuracy, and completeness. The principal investigator will supervise the research assistant/project coordinator in all data collection activities over the course of the study.

For guidance, refer to the following Partners policies:

Data and Safety Monitoring Plans and Quality Assurance
https://partnershealthcare-public.sharepoint.com/ClinicalResearch/DSMP_in_Human_Subjects_Research.pdf

Reporting Unanticipated Problems (including Adverse Events)

https://partnershealthcare-public.sharepoint.com/ClinicalResearch/Reporting_Unanticipated_Problems_including_Adverse_Events.pdf

PRIVACY AND CONFIDENTIALITY

Describe methods used to protect the privacy of subjects and maintain confidentiality of data collected. This typically includes such practices as substituting codes for names and/or medical record numbers; removing face sheets or other identifiers from completed surveys/questionnaires; proper disposal of printed computer data; limited access to study data; use of password-protected computer databases; training for research staff on the importance of confidentiality of data, and storing research records in a secure location.

NOTE: Additional measures, such as obtaining a Certificate of Confidentiality, should be considered and are strongly encouraged when the research involves the collection of sensitive data, such as sexual, criminal or illegal behaviors.

All information from individuals or entities in the course of this study that identifies an individual or entity will be treated as confidential in accordance with section 903c of the Public Health Service Act (42 U.S.C.299a-1). This will be done by keeping all personal identifiers in a separate location from the data, and only approved research personnel and study investigators trained in the protection of human subjects will have access to the linked data. All research staff will be properly trained in the importance of confidentiality of data.

All electronic data and files will be stored on a password-protected database in a shared file area (SFA) on a Partners password-protected computer behind the Partners firewall with anti-virus software or on Partners Research Computing approved cloud storage services. Data about consented patients will also be stored behind the RAND Corporation firewall in accordance with the terms of the consent. Patients' identifiers and other data collected on paper will be kept in locked filing cabinets. Data collection instruments used during the project and stored on laptop or desktop computers will also be password protected. Printed computer data with PHI will be shredded and disposed of upon completion of the study and any record-keeping requirements. Any identifiers will be removed prior to any analysis and all results will be presented in aggregate. The principal investigator will be responsible for the confidentiality and security of all study databases. These measures should be effective in preventing breaches of confidentiality.

SENDING SPECIMENS/DATA TO RESEARCH COLLABORATORS OUTSIDE PARTNERS

> Specimens or data collected by Partners investigators will be sent to research collaborators outside Partners, indicate to whom specimens/data will be sent, what information will be sent, and whether the specimens/data will contain identifiers that could be used by the outside collaborators to link the specimens/data to individual subjects.

No specimens or data will be sent to anyone outside of BWH.

> Specifically address whether specimens/data will be stored at collaborating sites outside Partners for future use not described in the protocol. Include whether subjects can withdraw their specimens/data, and how they would do so. When appropriate, submit documentation of IRB approval from the recipient institution.

Not applicable.

RECEIVING SPECIMENS/DATA FROM RESEARCH COLLABORATORS OUTSIDE PARTNERS

> When specimens or data collected by research collaborators outside Partners will be sent to Partners investigators, indicate from where the specimens/data will be obtained and whether the specimens/data will contain identifiers that could be used by Partners investigators to link the specimens/data to individual subjects. When appropriate, submit documentation of IRB approval and a copy of the IRB-approved consent form from the institution where the specimens/data were collected.

No specimens or data will be received from anyone outside of BWH.

Final Protocol

PRINCIPAL/OVERALL INVESTIGATOR

Ishani Ganguli MD MPH

PROTOCOL TITLE

A Multi-pronged Intervention to Initiate Shared Decision-making on Medical Tests and Care Cascades in the Primary Care Setting

FUNDING

Robert Wood Johnson Foundation

VERSION DATE

10/27/2022

SPECIFIC AIMS

Concisely state the objectives of the study and the hypothesis being tested.

Aims:

4. Develop a simple, scalable intervention to prompt conversations about the downstream consequences of potentially discretionary medical tests in the primary care setting.
5. Implement this email and text-based intervention in the primary care setting.
6. Rigorously evaluate the impact of this intervention using a randomized controlled trial study of 20 primary care physicians and at least 200 patients.

Hypotheses:

3. The intervention is feasible to implement in primary care practices with no major recruitment barriers as defined by recruiting 20 physicians from clinics affiliated with one academic medical center, and at least 200 patients of all genders and a range of ages, race/ethnicities, and education levels.
4. Intervention arm patients are more likely to have higher quality conversations about medical testing decisions (primary outcome).

BACKGROUND AND SIGNIFICANCE

Provide a brief paragraph summarizing prior experience important for understanding the proposed study and procedures.

Medical tests can have sizable downstream consequences including further tests, treatments, office visits, and even hospitalizations. Known as *care cascades*, these downstream services have significant direct and indirect cost implications for patients. In some cases, downstream services are medically appropriate, such as when an initial test is medically indicated and has a high degree of accuracy. However, many tests are performed even when they are not medically indicated (e.g., at a patient's request), and may have high rates of false positives or of incidental findings (i.e., unrelated to the purpose of the test). Notably, the following tests are commonly ordered, may be overused, and have a high risk of false positives and incidental findings (and therefore care cascades): imaging tests, electrocardiograms, and blood tests including blood count, electrolyte, kidney function, and liver function tests. Some estimates suggest that up to 52% of radiology and laboratory tests produce incidental findings, and that rate may increase with advances in technology.

Studies of specific cascades using national administrative claims data also suggest that these cascades can be costly – in one example by Ganguli et al, cascades following pre-operative electrocardiograms for cataract surgery cost ten times the initial electrocardiograms (ECGs). A recent national study of US internists conducted by Ganguli et al. also found that almost all responding physicians had experienced cascades after incidental findings that did not lead to clinically meaningful outcomes yet caused physical, psychological, or other harms to patients or the physicians themselves. Although some of these cascades may eventually reveal clinically important findings, more often they find nothing significant. This is especially true when the initial test is discretionary or even known to be of low-value, as in the example of pre-operative ECGs for cataract surgery. It is likely that few patients are aware of the potential for cascades or risk of false positives when making the decision with their clinicians to proceed with medical tests.

Despite the prevalence and burden of care cascades, little effort has been devoted to addressing them. Consideration of care cascades have not been incorporated into best practices for shared decision-making and we are not

aware of any interventions that have been developed or evaluated to educate patients or clinicians on the financial or psychological risk of care cascades.

RESEARCH DESIGN AND METHODS

Briefly describe study design and anticipated enrollment, i.e., number of subjects to be enrolled by researchers study-wide and by Partners researchers. Provide a brief summary of the eligibility criteria (for example, age range, gender, medical condition). Include any local site restrictions, for example, "Enrollment at Partners will be limited to adults although the sponsor's protocol is open to both children and adults."

Design: During Phase I, we will implement a user-centered design process to develop the educational content and implementation strategy for the intervention. Specifically, we will conduct focus groups with patients and clinicians in which we iteratively solicit feedback on the language and dissemination of text/email-based messages and interactive content for patients, on references and peer comparison emails for physicians, and on survey items for both.

In Phase II, we will randomize 20 primary care physicians to intervention and control arms, recruit at least 10 and up to 20 patients per physician with upcoming wellness visits, and implement our intervention. We will collect pre- and post-intervention surveys of patients and physicians, as well as post-study interviews of patients and physicians. In phase III, we will use mixed methods analysis to study the data collected in Phase II.

Enrollment: For Phase I, we will recruit up to 27 patients for the patient advisory committee and interviews and up to 20 practicing BWH primary care physicians for interviews.

For phase II, we will recruit 20 BWH primary care physicians who actively see patients and meet our inclusion criteria (based on patient-deidentified electronic health record data on test ordering rates during annual physicals). We will then recruit at least 10 patients for each of these PCPs. We will allow up to 20 patients per physician in the enrollment phase to ensure adequate sampling in case of loss to follow-up. All patients of the selected PCPs who are at least 18 years old, speak English, have access to email, and have not

opted-out of receiving research invitations from Mass General Brigham are eligible to participate.

Briefly describe study procedures. Include any local site restrictions, for example, "Subjects enrolled at Partners will not participate in the pharmacokinetic portion of the study." Describe study endpoints.

In phase I, we will develop a multi-pronged intervention for patients and clinicians. To this end, we will work with the BWH Center for Patients and Families to select and convene a 5-15 member patient advisory council. We will host two focus groups with this council, aided by discussion guides, to first identify key issues and then to suggest refinements to our intervention.

We will also recruit both patients and physicians for in-depth interviews. We will recruit up to 20 primary care physicians through the Patient-Based Research Network (PBRN) and suggestions from clinic medical directors. We will recruit up to 20 patients through the above Patient Advisory Council and the Rally platform. We will conduct up to 20 in-depth interviews each with these physicians and patients to understand their experiences with cascades and to cognitively test survey items.

Finally, we will review patient educational materials, clinician references, and clinician peer comparison emails with clinic leaders for their approval.

In phase II, we will work with clinic leaders in the BWH PBRN to recruit 20 physicians who have not previously been involved. We will select these physicians from among those who fall above the 25th percentile of test ordering rates during annual physicals. We will use matched pair randomization by testing rate and gender to randomize these PCPs to the control or intervention arms. For each physician, we will identify at least 10 of their patients who are scheduled for a physical in the next 6 months and meet our inclusion criteria. We will recruit them using patient portal messages.

Intervention arm: We will send emails to physicians with feedback on how they compare to their peers in aggregate on test ordering during annual

physicals, along with links to the physician-facing materials. We will not share identifiable data with a given physician on their peer physicians. One to two days before their visit, we will send patients educational materials via text and email.

Control arm: One to two days before their visit, we will send patients general information on visit preparation.

We will perform an exploratory chart review analysis of visits in the intervention and control arms to assess any documentation of discussion about medical testing and ordering of medical tests. Specifically, we will record physical visit note details on medical test conversations and medical tests ordered. This will help us understand if the intervention may have influenced ordering practices.

NOTE: We acknowledge that during the COVID-19 pandemic, clinical operations have changed drastically to protect both patients and health care workers. For instance, routine wellness visits have been cancelled or converted to virtual. Given our commitment to protect the safety and welfare of our study subjects and staff, and to avoid disrupting clinic operations during this challenging time, we plan to conduct phase I of our study virtually and to begin the intervention phase only once it is deemed safe and feasible to do so.

For studies involving treatment or diagnosis, provide information about standard of care at Partners (e.g., BWH, MGH) and indicate how the study procedures differ from standard care. Provide information on available alternative treatments, procedures, or methods of diagnosis.

Treatment and diagnosis are not part of this protocol.

Describe how risks to subjects are minimized, for example, by using procedures which are consistent with sound research design and which do not unnecessarily expose subjects to risk or by using procedures already being performed on the subject for diagnostic or treatment purposes.

There are no known risks to participating in this study. Patient and clinician subjects may voluntarily participate and withdraw at any time.

There is a theoretical risk of breach of data confidentiality and that PHI could become known to unauthorized persons, but we will take all steps necessary to protect PHI (see below). To mitigate this risk, we will follow all compliance and data confidentiality procedures for research at Partners. Specifically, we will use procedures consistent with sound research design and which do not expose subjects to unnecessary risk. Data monitoring will be conducted regularly (see below). All data analyzed will be de-identified and reported in aggregate.

> Describe explicitly the methods for ensuring the safety of subjects. Provide objective criteria for removing a subject from the study, for example, objective criteria for worsening disease/lack of improvement and/or unacceptable adverse events. The inclusion of objective drop criteria is especially important in studies designed with placebo control groups.

This is a minimal risk study. There is no known physical or medical safety threat to patient or clinician subjects who participate. Furthermore, participation is voluntary and all subjects will be informed that they may opt-out any time (see below). Given the minimal risk to subjects, our data safety and monitoring procedures, and the relatively small sample size, we do not plan on having drop criteria.

FORESEEABLE RISKS AND DISCOMFORTS

> Provide a brief description of any foreseeable risks and discomforts to subjects. Include those related to drugs/devices/procedures being studied and/or administered/performed solely for research purposes. In addition, include psychosocial risks, and risks related to privacy and confidentiality. When applicable, describe risks to a developing fetus or nursing infant.

For patient and clinician subjects, there are no known risks to participating in our data collection activities. There is a potential risk of physicians feeling psychological discomfort in knowing their test ordering rates. We will mitigate this by offering an option if they have concerns to speak with clinic leaders about these concerns. The main risks are breach of

confidentiality and privacy of information shared. Every effort will be made to maintain confidentiality and privacy of information shared and collected and subsequent data analysis (see below), and efforts will be made to minimize the duration of data collection activities.

EXPECTED BENEFITS

Describe both the expected benefits to individual subjects participating in the research and the importance of the knowledge that may reasonably be expected to result from the study. Provide a brief, realistic summary of potential benefits to subjects, for example, “It is hoped that the treatment will result in a partial reduction in tumor size in at least 25% of the enrolled subjects.” Indicate how the results of the study will benefit future patients with the disease/condition being studied and/or society, e.g., through increased knowledge of human physiology or behavior, improved safety, or technological advances.

Individual subjects may benefit by learning about (and, if appropriate, avoiding) possible downstream consequences of medical testing, and having their physicians become more informed about these consequences and how to discuss them. Future patients will benefit through improved knowledge of a possible intervention to facilitate patient-clinician conversations about cascades that may in turn help to reduce their negative effects.

EQUITABLE SELECTION OF SUBJECTS

The risks and benefits of the research must be fairly distributed among the populations that stand to benefit from it. No group of persons, for example, men, women, pregnant women, children, and minorities, should be categorically excluded from the research without a good scientific or ethical reason to do so. Please provide the basis for concluding that the study population is representative of the population that stands to potentially benefit from this research.

Our goal will be to enroll eligible patients whose characteristics are representative of the entire population of eligible patients. We will specifically target patients of all genders and a range of ages, race/ethnicities, and education levels.

When people who do not speak English are excluded from participation in the research, provide the scientific rationale for doing so. Individuals who do not speak English should not be denied participation in research simply because it is inconvenient to translate the consent form in different languages and to have an interpreter present.

We will approach English-speaking patients only. The scientific rationale for this limitation is because the intervention will be available in English in this

early stage of development. All clinician subjects caring for patients at BWH speak English.

For guidance, refer to the following Partners policy:
Obtaining and Documenting Informed Consent of Subjects who do not Speak English
https://partnershealthcare-public.sharepoint.com/ClinicalResearch/Non-English_Speaking_Subjects.1.10.pdf

RECRUITMENT PROCEDURES

Explain in detail the specific methodology that will be used to recruit subjects. Specifically address how, when, where and by whom subjects will be identified and approached about participation. Include any specific recruitment methods used to enhance recruitment of women and minorities.

For phase I, we will recruit the PAC and physician focus groups as described above.

For phase II, we will recruit physicians as described above. Eligible patient subjects will then be identified by research assistants trained in the protection of human subjects based on upcoming appointment using "minimum necessary" information in the EHR. To meet recruitment criteria, patients must not have opted out of receiving Mass General Brigham (MGB) research invitations. The research assistant will send the patient recruitment list and a letter created using the template provided by the MGB IRB to the DHeCare Research Team to create a letter project. Following project creation, the research assistant will send patients a letter through the patient portal. The letter includes an email address and phone number that allows patients to opt out. The letter also includes a link to REDCAP which will include an electronic consent form with information sheet, invitation to enroll, and the baseline survey.

Provide details of remuneration, when applicable. Even when subjects may derive medical benefit from participation, it is often the case that extra hospital visits, meals at the hospital, parking fees or other inconveniences will result in additional out-of-pocket expenses related to study participation. Investigators may wish to consider providing reimbursement for such expenses when funding is available

Patient subjects in both arms will be compensated $10 at the completion of the study. Physician subjects will be compensated $50 for the whole study.

For guidance, refer to the following Partners policies:

Recruitment of Research Subjects

https://partnershealthcare-public.sharepoint.com/ClinicalResearch/Recruitment_Of_Research_Subjects.pdf

Guidelines for Advertisements for Recruiting Subjects

https://partnershealthcare-public.sharepoint.com/ClinicalResearch/Guidelines_For_Advertisements.1.11.pdf

Remuneration for Research Subjects

https://partnershealthcare-public.sharepoint.com/ClinicalResearch/Remuneration_for_Research_Subjects.pdf

CONSENT PROCEDURES

Explain in detail how, when, where, and by whom consent is obtained, and the timing of consent (i.e., how long subjects will be given to consider participation). For most studies involving more than minimal risk and all studies involving investigational drugs/devices, a licensed physician investigator must obtain informed consent. When subjects are to be enrolled from among the investigators' own patients, describe how the potential for coercion will be avoided.

In phase I, eligible physician subjects will be informed about our research initiative via email and patients via RALLY. Prior to participation in interviews, we will confirm verbal consent. Specifically, these subjects will be informed that: 1) participation is voluntary–they may stop the interview at any time, and 2) data collected will be analyzed and reported in aggregate.

In phase II, potential patient subjects will be informed as part of the patient portal message and the REDCAP link that it will contain. This information will contain the purpose of the study, risks/benefits, methods of ensuring confidentiality, and voluntary nature of participation. Specifically, patient subjects will be informed that: 1) participation is voluntary–they may withdraw at any time; 2) a decision to not participate will in no way affect their care at BWH; 3) information shared will remain confidential and used only for research purposes; and 4) data collected will be analyzed and reported in aggregate. We will not request verbal consent as this will be

prohibitively burdensome for this minimal risk study and because this study does not share any personal health information with the patients.

NOTE: When subjects are unable to give consent due to age (minors) or impaired decision-making capacity, complete the forms for Research Involving Children as Subjects of Research and/or Research Involving Individuals with Impaired Decision-making Capacity, available on the New Submissions page on the PHRC website:

https://partnershealthcare.sharepoint.com/sites/phrmApply/aieipa/irb

For guidance, refer to the following Partners policy:

Informed Consent of Research Subjects:
https://partnershealthcare-public.sharepoint.com/ClinicalResearch/Informed_Consent_of_Research_Subjects.pdf

DATA AND SAFETY MONITORING

Describe the plan for monitoring the data to ensure the safety of subjects. The plan should include a brief description of (1) the safety and/or efficacy data that will be reviewed; (2) the planned frequency of review; and (3) who will be responsible for this review and for determining whether the research should be altered or stopped. Include a brief description of any stopping rules for the study, when appropriate. Depending upon the risk, size and complexity of the study, the investigator, an expert group, an independent Data and Safety Monitoring Board (DSMB) or others might be assigned primary responsibility for this monitoring activity.

NOTE: Regardless of data and safety monitoring plans by the sponsor or others, the principal investigator is ultimately responsible for protecting the rights, safety, and welfare of subjects under his/her care.

The principal investigator and research assistant/project coordinator will monitor data collection activities, maintain data integrity and quality control, protect the rights, safety, and welfare of study subjects, and adhere to standards set by the Partners IRB. All data will be stripped of PHI and electronic data will be kept on a secured shared file area (SFA) behind the Partners firewall with anti-virus software or Partners Research Computing approved cloud storage services. Data about consented patients will also be

stored behind the RAND Corporation firewall in accordance with the terms of the consent. Any paper forms and/or audio recordings will be destroyed after being transcribed into electronic format. Monitoring will be performed on a monthly basis during the study period and will include a review of enrollment, data analysis, and breaches of confidentiality, and any adverse events that may occur. Any adverse events will be graded as to their attribution to the intervention and reported according to IRB guidelines. The principal investigator will review any complaints reported from patient and physician subjects. These will be reported to the Partners IRB annually or immediately if the complaint is serious. Given the minimal risk of the study, we are not planning to use automatic stopping rules or a DSMB. We emphasize that this study does not involve any invasive procedures.

> Describe the plan to be followed by the Principal Investigator/study staff for review of adverse events experienced by subjects under his/her care, and when applicable, for review of sponsor safety reports and DSMB reports. Describe the plan for reporting adverse events to the sponsor and the Partners' IRB and, when applicable, for submitting sponsor safety reports and DSMB reports to the Partners' IRBs. When the investigator is also the sponsor of the IND/IDE, include the plan for reporting of adverse events to the FDA and, when applicable, to investigators at other sites.
>
> NOTE: In addition to the adverse event reporting requirements of the sponsor, the principal investigator must follow the Partners Human Research Committee guidelines for Adverse Event Reporting

Voluntary participation in data collection activities are not expected to cause adverse events to patient or physician subjects. However, if an adverse event occurs, the principal investigator will follow PHS HRC guidelines for adverse event reporting.

MONITORING AND QUALITY ASSURANCE

> Describe the plan to be followed by the principal investigator/study staff to monitor and assure the validity and integrity of the data and adherence to the IRB-approved protocol. Specify who will be responsible for monitoring, and the planned frequency of monitoring. For example, specify who will review the accuracy and completeness of case report form entries, source documents, and informed consent.
>
> NOTE: Regardless of monitoring plans by the sponsor or others, the principal investigator is ultimately responsible for ensuring that the study is conducted at his/her investigative site in

accordance with the IRB-approved protocol, and applicable regulations and requirements of the IRB.

The principal investigator and research assistant/project coordinator will monitor and assure the validity and integrity of the data collection and adherence to the procedures outlined in this protocol. They will review the study database on a monthly basis, ensuring data integrity, accuracy, and completeness. The principal investigator will supervise the research assistant/project coordinator in all data collection activities over the course of the study.

For guidance, refer to the following Partners policies:

Data and Safety Monitoring Plans and Quality Assurance
https://partnershealthcare-public.sharepoint.com/ClinicalResearch/DSMP_in_Human_Subjects_Research.pdf

Reporting Unanticipated Problems (including Adverse Events)
https://partnershealthcare-public.sharepoint.com/ClinicalResearch/Reporting_Unanticipated_Problems_including_Adverse_Events.pdf

PRIVACY AND CONFIDENTIALITY

Describe methods used to protect the privacy of subjects and maintain confidentiality of data collected. This typically includes such practices as substituting codes for names and/or medical record numbers; removing face sheets or other identifiers from completed surveys/questionnaires; proper disposal of printed computer data; limited access to study data; use of password-protected computer databases; training for research staff on the importance of confidentiality of data, and storing research records in a secure location.

NOTE: Additional measures, such as obtaining a Certificate of Confidentiality, should be considered and are strongly encouraged when the research involves the collection of sensitive data, such as sexual, criminal or illegal behaviors.

All information from individuals or entities in the course of this study that identifies an individual or entity will be treated as confidential in accordance with section 903c of the Public Health Service Act (42 U.S.C.299a-1). This will be done by keeping all personal identifiers in a separate location from the data, and only approved research personnel and study investigators trained in the protection of human subjects will have access to the linked data. All research staff will be properly trained in the importance of confidentiality of data.

All electronic data and files will be stored on a password-protected database in a shared file area (SFA) on a Partners password-protected computer behind the Partners firewall with anti-virus software or on Partners Research Computing approved cloud storage services. Data about consented patients will also be stored behind the RAND Corporation firewall in accordance with the terms of the consent. Patients' identifiers and other data collected on paper will be kept in locked filing cabinets. Data collection instruments used during the project and stored on laptop or desktop computers will also be password protected. Printed computer data with PHI will be shredded and disposed of upon completion of the study and any record-keeping requirements. Any identifiers will be removed prior to any analysis and all results will be presented in aggregate. The principal investigator will be responsible for the confidentiality and security of all study databases. These measures should be effective in preventing breaches of confidentiality.

SENDING SPECIMENS/DATA TO RESEARCH COLLABORATORS OUTSIDE PARTNERS

Specimens or data collected by Partners investigators will be sent to research collaborators outside Partners, indicate to whom specimens/data will be sent, what information will be sent, and whether the specimens/data will contain identifiers that could be used by the outside collaborators to link the specimens/data to individual subjects.

No specimens or data will be sent to anyone outside of BWH.

Specifically address whether specimens/data will be stored at collaborating sites outside Partners for future use not described in the protocol. Include whether subjects can withdraw their specimens/data, and how they would do so. When appropriate, submit documentation of IRB approval from the recipient institution.

Not applicable.

RECEIVING SPECIMENS/DATA FROM RESEARCH COLLABORATORS OUTSIDE PARTNERS

When specimens or data collected by research collaborators outside Partners will be sent to Partners investigators, indicate from where the specimens/data will be obtained and whether the specimens/data will contain identifiers that could be used by Partners investigators to link the specimens/data to individual subjects. When appropriate, submit documentation of IRB approval and a copy of the IRB-approved consent form from the institution where the specimens/data were collected.

No specimens or data will be received from anyone outside of BWH.

Statistical Analysis Plan

Measures

Outcomes: The primary outcome will be the Shared Decision-Making Process survey (SDMP_4 score), a 4 item measure that is validated as a patient-reported outcome performance measure. Each response will be scored as binary and added for a range of 0-4. Patient-level secondary outcomes will be presence of a testing discussion, satisfaction with the testing discussion, presence of a discussion of next steps, whether the doctor explained tests in a way that was easy to understand, and patient knowledge (a score of 0-4 based on correct responses to each of 4 knowledge survey items). Patient-level exploratory outcomes will include patients' survey responses about which tests were discussed, who raised the idea of tests, and the importance of various factors in testing decisions. Physician-level exploratory outcomes will include the importance of various factors in testing decisions, consideration of patient out-of-pocket costs in clinical decisions, self-reported discussion with patients about false positives, incidental findings, and cascades, and barriers to cascade conversations.

Among intervention group patients, we will use server log data to count how many viewed the website. This will produce a lower-bound estimate as some participants may copy-paste the link without the code to view the website, preventing us from tracking those views.

Physician characteristics: Time since residency, gender, race, ethnicity, time in outpatient practice, and prior experience with cascades (all from pre-study survey).

Patient characteristics: Age, gender, race, ethnicity, education (from pre-study survey), primary insurance (from electronic health record), time with PCP (from post-study survey), approach to medical action, decision-making preferences, and health literacy (from pre-study survey).

Quantitative Analysis

Our primary analysis will be Intention to Treat with multiple imputation of missing outcomes. We will summarize all data using simple descriptive statistics (means with standard deviations for continuous variables and frequencies with percentages for categorical variables) overall and in each arm. We will compare baseline characteristics between physicians in each group using t-tests and chi-square tests as appropriate and accounting for clustering by matched pair and by physician nested within matched pair. We will then summarize and compare baseline characteristics among 1) all enrolled patients in each group, 2) enrolled patients in each group with missing post-study surveys, and 3) enrolled patients in each group who completed post-study surveys (final sample), using t-tests and chi square tests as appropriate and clustering standard errors by matched pair and by physician nested within matched pair.

To estimate differences between intervention and control groups in our primary and secondary outcomes, we will use linear regression models (generalized estimating equations (GEE)) adjusted for patient age, gender, race/ethnicity, and education (because these covariates may be associated with the primary outcome) and any additional baseline covariates in the model that exhibit significant differences ($p<0.05$) between groups. We will also include standard errors clustered by matched pair and by physician nested within matched pair. Linear regression using

GEE to compare mean scores between treatment and control groups is robust to non-normality of the outcomes.

We estimate that we will have >80% power to detect a 0.5 standard deviation difference in the SDMP_4 measure between the intervention and control arms based on an expected sample of 200 patient-visits and a 2-sided type I error rate of 5% and assuming intra-cluster correlation coefficient (ICC) for patients from the same physician of 0.05.

Exploratory analysis of the primary outcome: To explore how the effect of the intervention on the primary and secondary outcomes varies within relevant population strata, we will repeat these analyses after stratifying patients by physician gender (male vs female), time with PCP (dichotomized), and health care preferences (wait and see vs taking action, make decisions vs defer decisions).

Analysis of patient-level exploratory outcomes – We will build GEE linear models with adjustment as described above.

Analysis of physician-level exploratory outcomes – We will use paired t-tests to compare intervention and control group physicians on pre-post differences in their responses to survey items on factors in testing decisions, consideration of patient out-of-pocket costs; self-reported discussion with patients about false positives, incidental findings, and cascades; and barriers to cascade conversations.

We will use R statistical software for data analysis and consider 2-sided P values to be significant at <0.05.

Qualitative Analysis

We will transcribe patient and physician interviews and will use an inductive thematic approach to analyze these qualitative data. Specifically, study authors will develop a code book, code the data using deductive (based on the interview guides) and inductive (based on categories that emerge during analysis) methods, resolve coding differences by consensus, and identify key categories and themes.
