# Supplementary material for: Effect of a Peer Comparison and Educational Intervention on Medical Test Conversation Quality: A Randomized Clinical Trial
Source: JAMA Netw Open. 2023 Nov 9;6(11):e2342464. doi: 10.1001/jamanetworkopen.2023.42464 (PMC10636635; doi:10.1001/jamanetworkopen.2023.42464)
Supplement: Supplement 3. — Data Sharing Statement [file jamanetwopen-e2342464-s003.pdf]

Data Sharing Statement

Ganguli. Effect of a Peer Comparison and Educational Intervention on Medical Test Conversation Quality. *JAMA Netw Open*. Published November 14, 2023.
doi:10.1001/jamanetworkopen.2023.42464

Data
Data available: Yes
Data types: Deidentified participant data, Data dictionary
How to access data: Requests for data should be sent to the corresponding author at iganguli@bwh.harvard.edu.
When available: With publication

Supporting Documents
Document types: None

Additional Information
Who can access the data: We will make the data available to researchers whose proposed use has been approved.
Types of analyses: Data will be made available for research purposes only.
Mechanisms of data availability: Data will be made available after approval of a proposal.
