# Supplementary material for: Effect of a Peer Comparison and Educational Intervention on Medical Test Conversation Quality: A Randomized Clinical Trial
Source: JAMA Netw Open. 2023 Nov 9;6(11):e2342464. doi: 10.1001/jamanetworkopen.2023.42464 (PMC10636635; doi:10.1001/jamanetworkopen.2023.42464)
Supplement: Supplement 2. — eMethods 1. Physician Peer Comparison Sample Email eMethods 2. Reference Materials for Intervention Group Physicians eAppendix 1. Physician Reference Materials on Interpretation of Medical Tests eAppendix 2. Physician Reference Materials on Incidental Findings eMethods 3. Physician Control Group Email eMethods 4. Reference Materials for Intervention Group Patients eMethods 5. Survey Instruments eMethods 6. Exit Interview Guides eTable 1. Baseline Patient Characteristics Stratified by Final Survey Completion Status eTable 2. Comparison of Patient-Reported Test Conversation Measures by Treatment Group eTable 3. Patient Responses to Individual Components of SDMP and Medical Test Knowledge Scores by Treatment Group eTable 4. Comparison of Patient-Reported Visit Descriptions and Factors Important to Testing Decisions by Treatment Group eTable 5. Summary of Physician Test Ordering Preferences and Behaviors, Prestudy and Poststudy, by Treatment Group eTable 6. Patient Analyses Stratified by Physician Gender and Patient Years With PCP eTable 7. Patient Analyses Stratified by Patient Preferences About Medical Decision-Making eTable 8. Baseline Characteristics of Interviewed Physicians and Patients eTable 9. Summary of Qualitative Patient Descriptions of Test Conversation During Study Visit eReferences [file jamanetwopen-e2342464-s002.pdf]

# Supplemental Online Content

Ganguli I, Mulligan KL, Chant ED, et al. Effect of a peer comparison and educational intervention on medical test conversation quality: a randomized clinical trial. *JAMA Netw Open*. 2023;6(11):e2342464. doi:10.1001/jamanetworkopen.2023.42464

**eMethods 1.** Physician Peer Comparison Sample Email

**eMethods 2.** Reference Materials for Intervention Group Physicians

**eAppendix 1.** Physician Reference Materials on Interpretation of Medical Tests

**eAppendix 2:** Physician Reference Materials on Incidental Findings

**eMethods 3.** Physician Control Group Email

**eMethods 4.** Reference Materials for Intervention Group Patients

**eMethods 5.** Survey Instruments

**eMethods 6.** Exit Interview Guides

**eTable 1.** Baseline Patient Characteristics Stratified by Final Survey Completion Status

**eTable 2.** Comparison of Patient-Reported Test Conversation Measures by Treatment Group

**eTable 3.** Patient Responses to Individual Components of SDMP and Medical Test Knowledge Scores by Treatment Group

**eTable 4.** Comparison of Patient-Reported Visit Descriptions and Factors Important to Testing Decisions by Treatment Group

**eTable 5.** Summary of Physician Test Ordering Preferences and Behaviors, Prestudy and Poststudy, by Treatment Group

**eTable 6.** Patient Analyses Stratified by Physician Gender and Patient Years With PCP

**eTable 7.** Patient Analyses Stratified by Patient Preferences About Medical Decision-Making

**eTable 8.** Baseline Characteristics of Interviewed Physicians and Patients

**eTable 9.** Summary of Qualitative Patient Descriptions of Test Conversation During Study Visit

**eReferences**

This supplemental material has been provided by the authors to give readers additional information about their work.

© 2023 Ganguli I et al. *JAMA Network Open.*

## eMethods 1. Physician Peer Comparison Sample Email

Subject    You are ordering more low-value tests than your top-performing BWH peers

Dear Dr. ________,

Thank you for agreeing to participate in this study.

Our analyses show that **you order more "routine" medical tests during physicals than top-performing primary care doctors at BWH Primary Care**.

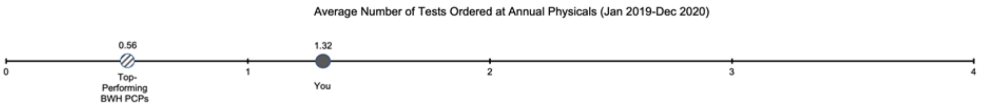


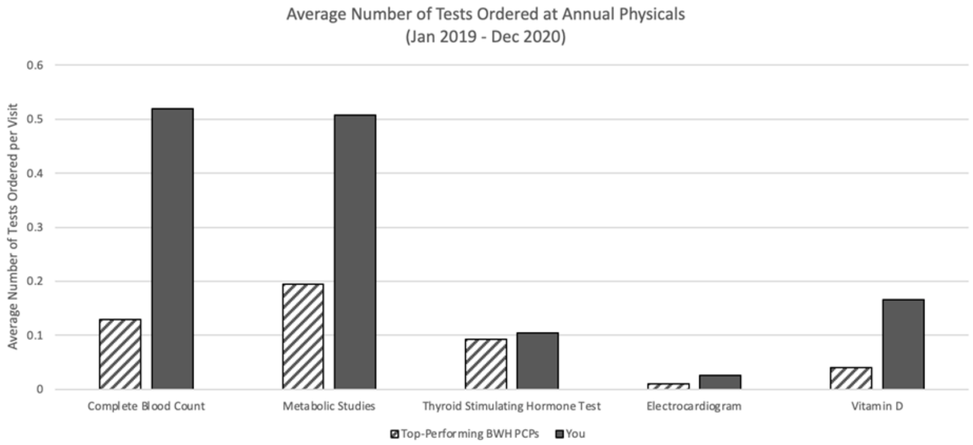


This information will not be shared with anyone else. These counts exclude physicals co-billed as problem-based visits; the counts also exclude tests ordered for specific conditions (e.g., CBC is not included if a patient has anemia on their problem list)."Metabolic Studies" refer to complete metabolic panel, liver function, and basic metabolic panel orders. "Top-performing" BWH primary care doctors are those ranked in the lower 50% of potentially low-value routine test ordering rates during annual physicals.

- **Do not order "routine" tests unless evidence supports them.** Research shows that routine blood counts, metabolic panels, urinalyses, and electrocardiograms are of little benefit in the absence of relevant symptoms or chronic conditions. Yet test results take us time to follow up, especially when there are borderline or incidental findings that may cause patients anxiety and prompt a cascade of phone calls, visits, tests, and treatments.
- **Use the attached PCOI guides** on interpreting test results and managing incidental findings.
- **Discuss the potential benefits and downsides of tests** during patient visits. We will send information on the basics of medical tests (also attached) to your patients with upcoming physicals who have agreed to be in our study.

**Additional resources:**

- Testing Wisely, interactive website for clinicians
- UpToDate: Evidence-based approach to prevention
- Washington Post article about cascades of care that can follow medical tests

Thank you for your commitment to providing outstanding care.

Sincerely,

BRIGHAM HEALTH BRIGHAM AND WOMEN'S HOSPITAL | Richard S. Gitomer, MD
Director, Primary Care Center of Excellence
P 617.525.9544

Ishani Ganguli, MD MPH

This email included weblinks to study physician reference materials (See Supplement Methods 2), study patient educational materials (See Supplement Methods 4), and other published resources (1–4).

© 2023 Ganguli I et al. *JAMA Network Open.*

## eMethods 2. Reference Materials for Intervention Group Physicians

Physician reference materials were developed through a user-centered design process consisting of multiple rounds of physician interviews and solicitation of reactions to drafted materials (5). The final materials included two references "Medical Test Interpretation" and "Incidental Findings" that were posted on Primary Care Office InSite (PCOI), an EHR-integrated point-of-care reference commonly used by health system primary care clinicians.

These PCOI materials for physicians included primers on statistical reasoning related to medical testing and scripted language and flowcharts designed to guide physicians through conversations with patients related to medical tests and incidental findings. Physician references can be found as Supplementary Materials 3 (Medical Test Interpretation) and Supplementary Materials 4 (Incidental Findings).

**eAppendix 1. Physician Reference Materials on Interpretation of Medical Tests**

**Keywords:**
***Authors:*** *Ishani Ganguli, MD, Shana Birnbaum, MD*
***Specialty Reviewer:*** *William Kormos, MD*

# Interpretation of Medical Tests

Background | Evaluation | Management| Resources | Patient Education | References

## Key Clinical Points

1. Patients may see test results on the patient portal as soon as they are completed. Discussing tests in advance (Table 1) may save patients and clinicians time, anxiety, and further medical services of uncertain value.
2. When deciding whether to order a medical test or interpreting the results, consider **pre-test probability** and **test characteristics** (including likelihood ratio, sensitivity, specificity, positive predictive value, and negative predictive value; (Figure 1).
3. Medical testing is most useful when the **pre-test probability of disease is intermediate** (e.g., 10-60%). When the pre-test probability is very low, the test gives little useful information and is more likely to generate false positives (Figure 2).

## Background

When considering medical tests and interpreting their results, it is important to consider their limitations and clearly communicate these limitations to patients.

- A test can result in a false positive (indicating the condition is present when it is not) or a false negative (indicating the condition is not present when it is).
- Both laboratory and imaging tests may reveal incidental findings of unclear significance (Supplement 4: Incidental Findings).
    - Biological systems exist on a continuum of values, yet laboratory results are often rounded to discrete integers or assigned thresholds to distinguish “normal” from “abnormal.” Reference ranges only capture 95% of true normal among studied populations who are thought to be healthy. This leaves 5% of healthy individuals outside of the “normal” range.
    - For many imaging tests, increased sensitivity has made “incidentalomas” more common.

As results are immediately visible to patients through their online portal, patients may see the results before their clinician has placed them in context. Incidental findings in these results may raise anxiety and lead to downstream tests and treatments (i.e., cascades) for uncertain gain. Therefore, it may be helpful to set patient expectations about medical testing *before* ordering tests to screen for conditions or to evaluate new

symptoms. **Table 1** presents a framework to consider when making and communicating testing decisions.

### Table 1: Framework for making decisions about medical testing

<table>
<tr><th colspan="2">Consider</th><th>Examples and scripted language</th></tr>
<tr><td colspan="2">What is the patient’s primary reason for presenting?</td><td>“What (diagnosis, outcome) are you concerned about?”<br>(For symptoms only) “Do you want to know what is causing the symptom(s), how to reduce or stop the symptom(s), or both?”</td></tr>
<tr><td rowspan="2">For symptoms only</td><td>How likely is it that the patient has a given disease now?<br>In other words, what is their pre-test probability based on community prevalence and personal risk factors?</td><td>“Based on your symptoms and your recent exposure, you likely have X.”</td></tr>
<tr><td>Will information from a test (or tests) change the diagnosis, management, or prognosis?<br>Consider pre-test probability and test characteristics to decide if a certain test will provide useful information. If pre-test probability for a condition is very low or very high, a test may not change what you do.</td><td>Diagnosis: This urinalysis and urine culture will tell us if your pain with urination is due to a urinary tract infection.<br><br>Management: This wound culture will help us pick the right antibiotic to treat your infection.<br><br>Prognosis: This rib x-ray will help us know if you have a broken rib; this will not change how we treat your pain, but it will help us predict how long it might last.</td></tr>
<tr><td colspan="2">Is the potential benefit from this information greater than the potential harm from the test(s)?<br>Consider harms such as patient time, inconvenience, and physical discomfort, as well as clinician time and effort to follow up.</td><td>If benefits < harms:<br>“This test will not provide useful information.”<br>“The potential for harm outweighs the potential for benefit.”<br>“Fortunately, you don’t need screening tests this year.”</td></tr>
<tr><td colspan="2">What are the next steps?</td><td>If ordering test(s):<br>“If the test shows X, we’ll do Y.”<br>If not ordering test(s):<br>“Let’s watch and wait / use the test of time. We will follow up on [date].”<br>“I hear that you want to do something for your health. Here’s what can help. Let’s focus on [relevant approach to promote health].”</td></tr>
</table>

© 2023 Ganguli I et al. *JAMA Network Open.*

## Evaluation

### Test Characteristics and Interpretation

To interpret test results, consider the pre-test probability of a given diagnosis and the test characteristics. **Figure 1** summarizes these test characteristics.

**Figure 1. Test Characteristics**

| | | True History of Disease | |
|---|---|---|---|
| **Test** | | + | - |
| | + | TP (True positive) | FP (False positive) |
| | - | FN (False negative) | TN (True negative) |
| **Community prevalence** | | Number in community WITH | Number in community WITHOUT |

- **Sensitivity (SE) -** The "true positive rate." In other words, the proportion of those who truly have a condition who are correctly identified by the test. This value is determined by the test alone.
  - **SE = TP / TP + FN**

- **Specificity (SP) –** The "true negative rate." In other words, the proportion of those who truly do not have the condition who are correctly identified by the test. This value is determined by the test alone.
  - **SP = TN / FP + TN**

- **Positive predictive value (PPV) –** The proportion of positive tests that are true positives. That is, the probability that a patient who has a positive test truly has the condition. This value depends on the test's sensitivity and specificity as well as the community prevalence of the disease.
  - **PPV = TP / TP + FP**

- **Negative predictive value (NPV) –** The proportion of negative tests that are true negatives. That is, the probability that a patient who has a negative test truly does not have the condition. This value depends on the test's sensitivity and specificity, as well as the community prevalence of the disease.
  - **NPV = TN / TN + FN**

To decide if a diagnostic test is needed, and which test to use, consider:

- **Pre-test probability:** Probability of a patient having a given condition before the diagnostic test result is known; based on the community prevalence of the condition and the patient's clinical presentation.

© 2023 Ganguli I et al. *JAMA Network Open.*

- **Likelihood ratio (LR):** For a given diagnostic test, the ratio of the probability that a result is correct to the probability that the result is incorrect.

- **Positive likelihood ratio (LR+)** = the likelihood that a positive result is correct.
    - **LR+ = SE / 1 - SP**
    - **True positive rate / False positive rate**

- **Negative likelihood ratio (LR-)** = the likelihood that a negative result is correct.
    - **LR- = 1 - SE / SP**
    - **False negative rate / True negative rate**

- **Post-test probability:** Probability of a patient having a given condition after the diagnostic test result is known; based on the pre-test probability, test result, and likelihood ratio. According to Bayes' Theorem, post-test odds = pre-test odds x LR. Calculate using the Fagan nomogram (Figure 2).

The **Bayes medical calculator** provides an online interactive calculator to help determine predictive values and likelihood ratios.

The Fagan nomogram (Figure 2) demonstrates the relationship between pretest probability of having a condition, the likelihood ratio of the test, and the post-test probability of that condition. Draw a straight line from the estimated pretest probability to the likelihood ratio for the given test, then continue the line to determine how a positive test result would change the probability of the condition post-test.

Most tests that are clinically "useful" have LR+ of >5 or LR- <0.2. Strong tests are >10 and <0.1. A likelihood ratio of 1 is a useless test (true positive = false positive, a coin flip) and tests in the 0.5 to 2 range are usually unhelpful. A test may be strong in one direction and not the other (e.g., D-Dimer has a poor (low) LR+ but a strong (low) LR-).

The nomogram shows that if there is very low or very high pretest probability, a test is unlikely to change the probability of having the condition.

© 2023 Ganguli I et al. *JAMA Network Open.*

**Figure 2. Fagan nomogram**

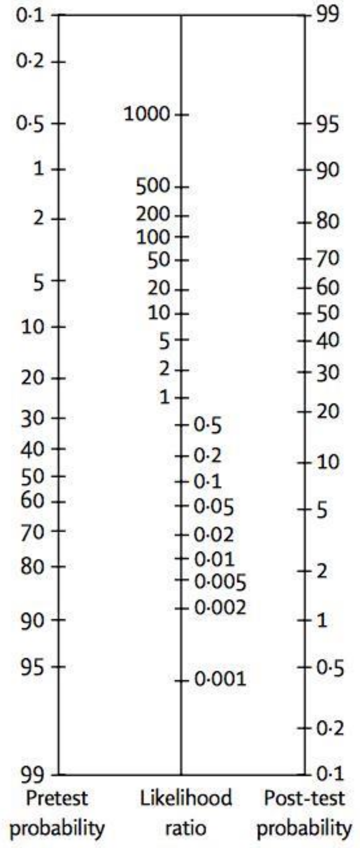


## Examples

### *(1) Mammogram for an average risk woman in her 40s*[1]

The patient is a woman in her 40s who is considering getting a mammogram.

- Assume test sensitivity 77-95% and specificity 94-97%.
- Assume prevalence of breast cancer 0.25% among 40-49-year-olds.[1]
- Using the Bayesian calculator, PPV is 3-9% and NPV is <0.05%.

*"If you take 100 similar women with positive mammograms, 3 to 9 mammograms out of 100 would be true positives while 91 to 97 mammograms would be false positives."*

### *(2) Exercise stress electrocardiogram test with moderate pre-test probability for coronary artery disease*

The patient is a man in his 50s with hypertension, hyperlipidemia, diabetes, and active smoking who presents with atypical chest pain.

- Assume test sensitivity 68% and specificity 77%.[2]
- Assume pre-test probability of coronary artery disease is 47%.[3]
- Using the Bayesian calculator, PPV is 72% and NPV is 73%.

© 2023 Ganguli I et al. *JAMA Network Open.*

*“If you take 100 similar men with positive stress tests: 72 out of 100 tests would be true positives and 28 would be false positives. If you take 100 similar men with negative stress tests: 73 out of 100 would be true negatives and 27 would be false positives.”*

Additional examples of clinical rules that use pre-test probability, sensitivity, and specificity to guide management include:

- Centor clinical criteria for Strep pharyngitis
- Ottawa ankle rule
- Ottawa knee rule
- Wells criteria for venous thromboembolism

## Resources

For more interactive tools on interpreting test results, visit ***www.testingwisely.com.***

## Patient education

- Medical Testing: The Basics (English)

## References


1. Morgan DJ, Pineles L, Owczarzak J, et al. Accuracy of Practitioner Estimates of Probability of Diagnosis before and after Testing. *JAMA Intern Med*. Published online 2021. doi:10.1001/jamainternmed.2021.0269
2. Bourque JM, Beller GA. Value of exercise ECG for risk stratification in suspected or known CAD in the era of advanced imaging technologies. *JACC Cardiovasc Imaging*. 2015;8(11):1309-1321. doi:10.1016/j.jcmg.2015.09.006
3. Pre-test probability of CAD (CAD consortium) | QxMD. Accessed June 16, 2021. https://qxmd.com/calculate/calculator_287/pre-test-probability-of-cad-cad-consortium#



*This material was created as part of a study funded by the Robert Wood Johnson Foundation. It was developed using literature review, content expert review, and interviews with BWH primary care physicians.*



© 2023 Ganguli I et al. *JAMA Network Open.*

**eAppendix 2. Physician Reference Materials on Incidental Findings**

**Keywords:**
***Authors:*** *Ishani Ganguli, MD, Shana Birnbaum, MD*
***Specialty Reviewers:*** *Anand Dighe, MD, Reece Goiffon, MD, William Kormos, MD, William Mehan, MD, Jo-Anne Shepard, MD*

# Incidental Findings

Background | Evaluation | Management| Resources | Patient Education | References

## Key Clinical Points

4. Incidental findings are common and may lead to cascades of further tests and treatment that are of uncertain value.
5. Consider the potential for incidental findings when ordering tests (see Supplement 4: Medical Test Interpretation) and use evidence to guide management of the incidental findings.
6. Common laboratory tests (ie, CBC [Table 1], BMP/CMP [Table 2], Urinalysis [Table 3]) may show results outside the normal range that are of unclear significance.
7. Imaging tests are increasingly sensitive and often detect incidental findings (Table 4). **Table 6** reviews management of some common incidental findings.

## Background

**Incidental findings** are abnormal or unexpected test results on laboratory and imaging tests that are unrelated to why the test was ordered. Though incidental findings are often benign or clinically insignificant, they can cause clinician and patient anxiety and spur cascades of downstream tests and treatments with uncertain benefits and possible harms. Patients can now see results immediately through their online portal, usually before their clinician has placed the results in context. They may seek alternative, potentially misleading, sources to help interpret results.

When discussing incidental findings with patients, consider framing the results as follows:
*“These are common – like freckles - and will not cause harm.”*
*“There is no such thing as zero risk; doing more tests also has tradeoffs.”*
*“You don’t need to worry about this.”*
*“The best next step is the test of time.”*
*“We’ll keep our eye on this; we’ll repeat it.”*
*“In isolation, this doesn’t mean anything bad for your health.”*
*“There is no need for concern.”*

© 2023 Ganguli I et al. *JAMA Network Open.*

## Evaluation

### Incidental Findings on Laboratory Tests

Test results may fall outside the stated normal range without fitting a meaningful clinical pattern (e.g., a slightly elevated MCV on an otherwise normal complete blood count in a healthy person). For most laboratory test results, the reference range captures 95% of true normal among studied populations. So rather than reflecting a clinically significant abnormality, a result that is "out of range" may instead reflect:

- A physiologic variant in the top or bottom 2.5% of the normal distribution.
- When there are multiple tests or test components, a result that is abnormal by statistical chance alone.
- A physiologic variant in a member of a population that is underrepresented in studies (e.g., minority groups, women, children, older adults).
- A normal physiologic variant because the clinical context in which the test was performed did not match the range provided by the lab (e.g., a non-fasting glucose >100 flagged as abnormal because the range assumes a fasting state).
- An abnormal result that does not fit a meaningful clinical pattern.

**Tables 1-3** review interpretation of results from common laboratory tests. To address an incidental result of potential significance, consider repeating the laboratory test.

**Table 1. Interpretation of complete blood count with differential**

| Components | Causes of low | Causes of high | Interpretation |
|---|---|---|---|
| White blood cell count (WBC) | Physiologic variant, bone marrow disorders/damage, lymphoma, autoimmune diseases, nutritional deficiency, sepsis, immune deficiency diseases like HIV. | Infection, inflammation, leukemia, necrosis (trauma, burns, surgery, etc.) allergies, steroid use, cigarette smoking (common), stress/ exercise, obesity. | Total number of WBC in sample. |
| Red blood cell count (RBC) | Trauma/bleeding (acute or chronic), RBC destruction, nutritional deficiency (e.g. deficiency of iron, folate, B12, or B6), bone marrow damage/disorders, kidney disease/failure, chronic inflammatory disease. | Dehydration, pulmonary disease, congenital heart disease, erythropoietin-producing tumor in kidneys, smoking, high altitudes, alpha and beta thalassemic trait, polycythemia vera. | Total number of RBC in sample. Interpreted with Hgb and Hct. |
| Hemoglobin (Hgb) and Hematocrit (Hct) | Similar causes as low RBC count, thalassemia (if high RBC), pregnancy. | Similar causes as high RBC count, dehydration is most common. | Hemoglobin = total amount of hemoglobin in blood. Hematocrit = Percentage of blood volume made of RBC. |
| Mean corpuscular (MCV) | Iron deficiency, thalassemia, anemia of chronic disease, hemoglobinopathies including hemoglobin C trait. | Vitamin B12 or folate deficiency, medications that impact B12/folate pathways, other medications including Zidovudine and Phenytoin, myelodysplastic syndrome, liver disease, hypothyroidism, alcohol use. | Average size of RBC in sample. It is interpreted along with RBC count to determine causes of abnormal results. |

© 2023 Ganguli I et al. *JAMA Network Open.*

| | | | |
|---|---|---|---|
| Mean corpuscular hemoglobin (MCH) | Similar causes as low MCV count. | Similar causes as high MCV count | Average amount of hemoglobin in each RBC. Results often reflect MCV results. |
| Mean corpuscular hemoglobin concentration (MCHC) | Primarily iron deficiency, less commonly decreased in thalassemia. | Autoimmune hemolytic anemia, cold agglutinins (artifactual), in burn patients, hereditary spherocytosis. | Average concentration of hemoglobin per volume of cell. |
| Red blood cell distribution width | Low variation (uniformity) in RBC size. | Wide variation in RBC size. Iron or vitamin B12 or folate deficiency, thalassemia (usually mild in trait). | Measures variation in RBC volume. |
| Platelet count | Viral infections (mononucleosis, hepatitis, HIV, or measles), rocky mountain spotted fever, platelet autoantibody, many drugs including acetaminophen, quinidine sulfa drugs, and heparin, cirrhosis, autoimmune diseases, sepsis, leukemia, lymphoma, myelodysplasia, chemotherapy or radiation, DIC, TTP, HUS. | Cancer (commonly lung, GI, ovarian, breast, or lymphoma), inflammatory diseases (e.g., Celiac disease, vaculitides), iron deficiency, hemolytic anemia, infections (e.g., tuberculosis) myeloproliferative disorder, exercise, allergic reactions, medication reactions (e.g., hormonal contraceptive pills), functional and surgical asplenia, tissue damage (e.g., trauma, acute pancreatitis, post-surgical period). | Total number of platelets in sample. |
| Mean platelet volume (MPV) | Issue with platelet production. Low result signifies the presence of older platelets. | Conditions that increase platelet production including immune thrombocytopenia and myeloproliferative disorders. High result signifies more young platelets. | Average size of platelets in sample. |
| White blood cell differential | *Neutrophils* – myelodysplastic syndrome, sepsis, nutritional deficiencies, drug reactions, bone marrow disorders/diseases, autoimmune diseases, cancer in bone marrow, congenital neutropenia.<br><br>*Lymphocytes* – autoimmune disorders, infections from HIV, TB, hepatitis, influenza, and COVID-19, bone marrow damage, corticosteroids.<br><br>*Monocytes* – bone marrow disease/damage, hairy cell leukemia, aplastic anemia.<br><br>*Eosinophils* – Almost always absent after administration of high dose corticosteroids. | *Neutrophils* – acute bacterial infection, inflammation, necrosis (trauma, burns, surgery), physiological stress, corticosteroids, 3rd trimester pregnancy, chronic myeloid leukemia, Cushing syndrome.<br><br>*Lymphocytes* – acute viral and bacterial infections, toxoplasmosis, chronic inflammatory disorders, lymphocytic leukemia, lymphoma, acute stress.<br><br>*Monocytes* –chronic infections (eg, TB), infections in the heart, collagen vascular diseases, monocytic | The number of specific types of WBCs.<br><br>For monocytes, one low reading is generally not significant.<br><br>Eosinophils and basophils generally have a low count, so low numbers are not significant. |

© 2023 Ganguli I et al. *JAMA Network Open.*

| | | | |
|---|---|---|---|
| | | leukemia, myelomonocytic leukemia.<br><br>*Eosinophils* –asthma, allergies, drug reactions, skin inflammation, parasitic infection, inflammatory disorders, Hypereosinophilic myeloid neoplasms, Addison disease, connective tissue disorders.<br><br>*Basophils* – allergic reactions (hives or food allergies), inflammation, some leukemias, uremia. | |

***Source:*** *https://labtestsonline.org/tests/complete-blood-count-cbc*
This list is not exhaustive.

**Table 2. Interpretation of comprehensive metabolic panel**

| Components | Causes of low | Causes of high | Interpretation |
|---|---|---|---|
| Sodium | Diarrhea, vomiting, excessive sweating, drinking too much water, diuretics, kidney disease, Addison's disease, edema from heart failure or cirrhosis, and excess ADH production, hyperglycemia, exogenous solutes, pseudohyponatremia. | Dehydration, Cushing syndrome, or diabetes insipidus. | Interpreted along with other electrolytes. |
| Potassium | Diarrhea/vomiting, alcoholism, Conn syndrome, acetaminophen overdose, poorly-controlled diabetes after taking insulin, potassium-wasting diuretics, and several other drugs. | Hemolysis induced during phlebotomy, dehydration, potassium-supplements, IV fluids, renal failure, tissue injury, infection, kidney disease, Addison's disease, diabetes, medications (potassium-sparing diuretics, NSAIDS, beta blockers, ACE inhibitors), pseudohyperkalemia. | Interpreted along with other electrolytes. |
| Chloride | Conditions that cause low sodium, along with metabolic alkalosis and respiratory acidosis. | Dehydration (most common), conditions that cause high sodium. | Results generally mirror sodium levels. |
| CO2 | Addison's disease, diarrhea, diabetic ketoacidosis, metabolic acidosis, respiratory alkalosis, shock, kidney disease, ethylene glycol/methanol poisoning, aspirin overdose. | Severe vomiting/diarrhea, lung diseases, Cushing syndrome, Conn syndrome, metabolic alkalosis. | Measure of total $CO_2$ in the blood and blood pH. Blood is normally slightly basic. |
| Anion gap | Low albumin, paraproteinemia. | Metabolic acidosis, laboratory error/prolonged transit. | Calculated value of sodium minus chloride and CO2. |
| BUN | Rarely concerning. Can be found in patients with severe liver disease or malnutrition. | Dehydration, increased protein diet, kidney disease/damage, kidney stones, or conditions with decreased blood flow to | Levels within normal range signify proper kidney function. |

| | | | |
|---|---|---|---|
| | | the kidneys, upper gastrointestinal bleed. | Interpreted along with creatinine. |
| eGFR | eGFR below 60 mL/1.73m$^2$ for >3 months, along with a marker of kidney damage, indicates chronic kidney disease. Stage of chronic kidney disease depends on the eGFR range (e.g., eGFR below 15 mL/1.73m$^2$ signifies CKD Stage 5). There may be expected, age-related decline in eGFR among adults aged ≥70. eGFR may underestimate renal function among older adults with higher than average muscle mass. | N/A | Calculated from creatinine, age, and sex. |
| Creatinine | Malnutrition, low muscle mass. | Acute or chronic kidney disease, infection, autoimmune disease, tubular necrosis caused by drugs or toxins, kidney stones, or conditions with decreased blood flow to the kidneys, rhabdomyolysis. | Interpreted along with eGFR. |
| Glucose | Starvation and use of glucose-lowering products. Rarely adrenal insufficiency, excessive drinking, liver disease, hypopituitarism, hypothyroidism, severe infections, severe heart failure, chronic kidney failure, insulinomas. Low glucose can be artifactual in samples collected in the incorrect tube or with long delays in sample processing. | Non-fasting sample, diabetes, acute stress. Rarely acromegaly, Cushing syndrome, hyperthyroidism, pancreatitis, or pancreatic cancer. | Presented normal range is based on fasting glucose only. |
| Calcium | Most commonly due to low blood protein levels (like albumin), chronic renal failure, low vitamin D. | Hyperparathyroidism or bone metastasis of carcinoma of breast, prostate, thyroid, or lung. | |
| Liver function tests<br>Also see: PCOI guideline: Evaluation of Abnormal Liver Tests | | | |
| Alkaline Phosphatase | Temporarily low ALP can be caused by blood transfusions or heart bypass, but persistent low levels could signify hypophosphatasia. Rarely, Wilson's disease. | High ALP usually indicates liver damage/disease, cholestasis, bone diseases like Paget's disease, or hyperparathyroidism. | Enzyme bound to hepatic canalicular membrane; also found in bone, intestine, placenta, kidney, leukocytes, and some neoplasms. |
| ALT | Marker for frailty | Liver disease, e.g. hepatitis or cirrhosis. | More specific to the liver than AST, but can also rise in acute skeletal muscle injury. |
| AST | N/A | Liver disease, e.g. hepatitis, cirrhosis, or certain liver cancers. Rhabdomyolysis. Strenuous exercise, acute pancreatitis, muscle disease, and heart attacks. | Less specific to the liver than ALT, can also rise in acute skeletal muscle injury. A high AST/ALT ratio suggests increased |

© 2023 Ganguli I et al. *JAMA Network Open.*

| | | | |
|---|---|---|---|
| | | | levels may be from another source such as the heart or skeletal muscle. |
| Total bilirubin | N/A | *Unconjugated bilirubin:* Gilbert syndrome, RBC destruction, or liver disease. *Conjugated bilirubin:* liver disease and conditions with bile duct blockages, like gallstones, tumors, and scarring of the bile ducts. | Sum of direct and indirect bilirubin. |
| Albumin | Liver or kidney disease, inflammation/acute illness, shock, malnutrition, Crohn's, Celiac disease, infections, burns, surgery, cancer, diabetes, hypothyroidism, and carcinoid syndrome. | Dehydration. | Marker of hepatic synthetic function; half-life of albumin is 20 days so decreases slowly in response to hepatic injury |
| Total Protein | Liver or kidney disease, malnutrition, celiac disease and irritable bowel disease. | Chronic inflammation/infections or bone marrow disorders, dehydration, MGUS/multiple myeloma. | Interpreted along with albumin. |

*Sources: UpToDate,* [*https://labtestsonline.org/tests/comprehensive-metabolic-panel-cmp*](https://labtestsonline.org/tests/comprehensive-metabolic-panel-cmp)
This list is not exhaustive.

**Table 3. Interpretation of urinalysis**

| Components | Interpretation |
|---|---|
| Glucose | Not normally present in urine. Signifies excess blood glucose or a decreased blood glucose threshold concentration. It can indicate diabetes, hormonal disorders, liver disease, or pregnancy. |
| Ketones | Not normally present in urine. Signifies fat metabolism and a decreased availability of carbohydrates. This can be a result of fasting, starvation, high-protein diets, exercise, cold exposure, frequent vomiting, or digestive system diseases. In people with diabetes, may suggest insufficient insulin and diabetic ketoacidosis. |
| Specific gravity | Signifies urine concentration. High specific gravity shows more concentrated urine and may be a sign of dehydration. Radiology contrast agents increase specific gravity for subsequent 24-48 hours. |
| Blood | Normally present in urine in low concentrations. Only abnormal if >3 RBCs.<br>A positive blood dipstick test indicates hematuria, hemoglobinuria, or myoglobinura. Urine microscopy should be used to confirm the diagnosis of hematuria. A positive "blood" dipstick result with no RBCs present may suggest presence of hemoglobin (due to RBC breakdown, for example if there is delayed analysis of the urine sediment) or myoglobin from muscle injury. |
| pH | Indicates acid-base status. Urine is normally slightly acidic. Affected by any condition that produces acids or bases in the body, such as acidosis or alkalosis, or by ingestion of acidic or basic foods. Urine pH affects kidney stone formation and can be modified through diet or drugs to reduce formation. |
| Protein | Normally not present or present in low concentrations. Causes include kidney disease, dehydration, stress, exercise, fever, aspirin therapy, and exposure to cold. |
| Bilirubin | Not normally present in urine. Signifies excess conjugated bilirubin eliminated through urine, can indicate biliary obstruction or hepatitis. Interpret along with urobilinogen. |
| Urobilinogen | Normally present in urine in low concentrations. Urobilinogen result from intestinal metabolism of bilirubin, so normal urobilinogen with high urine bilirubin suggests biliary obstruction. |

© 2023 Ganguli I et al. *JAMA Network Open.*

| | |
|---|---|
| | Positive result may suggest liver disease (hepatitis, cirrhosis, acute liver injury) or increased RBC destruction. Interpret along with bilirubin. |
| Nitrite | Not normally present in urine. Signifies bacteria in the urinary tract and indicates a urinary tract infection. Not all bacteria produce nitrite so may be negative even if patient has a urinary tract infection. Interpret along with leukocyte esterase.<br>A positive urine dipstick (either leukocyte esterase or nitrite positive) has a sensitivity of 75% and specificity of 82% for diagnosing a UTI, can lead to undertreatment when pre-test probability high. |
| Leukocyte Esterase | A positive result signifies excess white blood cells in the urine and can indicate inflammation of the urinary tract or kidneys from an infection. Interpret along with nitrite.<br>A positive urine dipstick (either leukocyte esterase or nitrite positive) has a sensitivity of 75% and specificity of 82% for diagnosing a UTI, can lead to undertreatment when pre-test probability high. In elderly, low specificity. Trace results usually not meaningful. |

*Sources: UpToDate, https://labtestsonline.org/tests/urinalysis*
This list is not exhaustive.

## Incidental Findings on Imaging Tests

"Incidentalomas" such as a renal cyst on abdominal CT scan have become increasingly common as the use and sensitivity of medical technology grows.

**Table 4** shows the prevalence of incidental findings on common imaging tests, **Table 5** shows the percentage of malignant incidentalomas by organ, and **Table 6** provides information on the management of commonly discovered incidental findings.

**Table 4. Incidental finding rates for common imaging tests**

| Imaging test | Organ(s) of incidentaloma | Prevalence of incidentalomas |
|---|---|---|
| Brain MRI | Brain | 22% |
| Spine MRI | Spine | 22% |
| Cardiac MRI | Extra-cardiac | 34% |
| CT chest | Thorax, abdomen, spine, heart | 45% |
| | Pulmonary vasculature | 2% |
| CT colonoscopy | Extra-colon | 38% |
| PET, PET/CT | Thyroid, colon, parotid, breast, prostate | 0.25-35% |

*Source: O'Sullivan, et al,[1] Britt, et al[2]*

**Table 5. Percentage of incidentalomas that were malignant, by organ**

| Organ of incidentaloma | Proportion of malignancies |
|---|---|
| Adrenal | 0.0007% |
| Ovary | 28% |
| Brain | 0% |
| Breast | 42% |
| Extra-colonic | 14% |
| Renal | 25% |
| Thyroid (asymptomatic, no history of cancer) | 28% |
| Thyroid* | 19% |
| Colon* | 17% |
| Parotid* | 5% |
| Prostate* | 11% |

Source: *O'Sullivan, et al,[1] *Included patients with cancer*

## Table 6. Guidance on common incidental findings

<table>
<tr><th>Incidental finding</th><th>Imaging</th><th>Guidance</th></tr>
<tr><td>Brain findings</td><td>Brain MRI, head CT</td><td>**Prevalence:** In meta-analysis, 2.0% of brain MRI recipients had **non-neoplastic incidental brain findings** including cyst (colloid 0.04%, arachnoid 0.50%), structural vascular abnormality (aneurysm: 0.35%), demyelination (0.09%), hydrocephalus (0.10%), Arnold-Chiari malformation (0.24%), and extra-axial collection (0.04%).<br>0.7% had **neoplastic incidental brain findings** including meningioma (0.29%), pituitary adenoma (0.15% PCOI guideline: Pituitary Adenoma) low grade glioma (0.05%), acoustic neuroma (0.03%), lipoma (0.04%), epidermoid tumor (0.03%), or unspecified neoplasm (0.09%).<br>Prevalence of these findings as well as of silent infarcts and white matter hyper-densities increase with age:<br>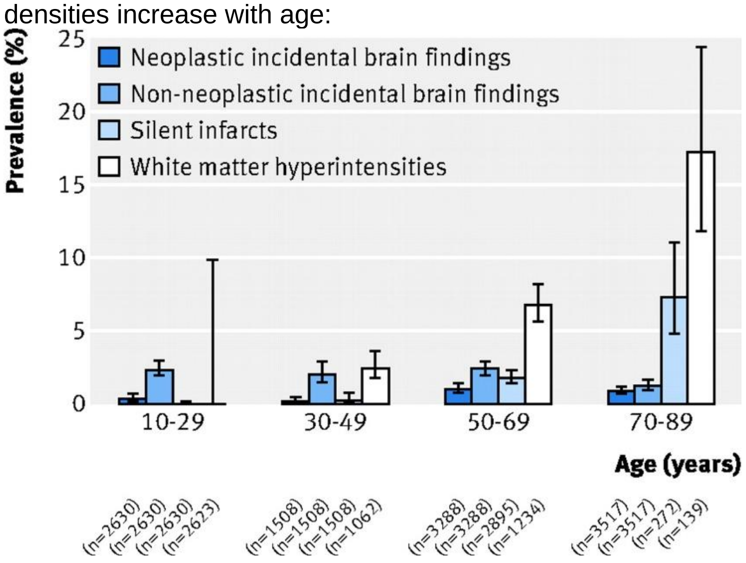

<br>Source: Morris, et al.[3]</td></tr>
<tr><td>Spine degenerative disc disease</td><td>CT, MRI of chest, abdomen, pelvis, spine</td><td>**Prevalence:** Common spine imaging finding, only sometimes associated with back pain.<br>*Rate of disk degeneration seen on imaging in asymptomatic individuals:*
<table>
<tr><td>Age, y</td><td>%</td></tr>
<tr><td>20s</td><td>37</td></tr>
<tr><td>30s</td><td>52</td></tr>
<tr><td>40s</td><td>68</td></tr>
<tr><td>50s</td><td>80</td></tr>
<tr><td>60s</td><td>88</td></tr>
<tr><td>70s</td><td>93</td></tr>
<tr><td>80s</td><td>96</td></tr>
</table>
Source: Brinjikji, et al[4]</td></tr>
<tr><td>Thyroid nodules</td><td>Ultrasound, CT</td><td>**Prevalence:** 20-70%. More common in women and older adults.<br>**Symptoms:** Most are asymptomatic and non-functional. When present, symptoms can include dysphagia, dysphonia, pressure, and pain.<br>**Prognosis:** 7-15% of those 1 cm or larger are malignant.<br>**Next Steps:** Thyroid function test to detect hyper/hypothyroidism. Further follow-up based on risk assessment. See PCOI guideline: Thyroid Nodules.</td></tr>
<tr><td>Pulmonary nodules</td><td>X-ray, CT, MRI</td><td>Defined as nodules that are <3 cm in size.<br>**Prevalence:** 8-51% of patients undergoing lung cancer screening<br>**Next Steps:** Fleischner criteria (specific for adults 35+ without active cancer in past 5 years) to assess probability of malignancy with clinical features (age, smoking history, hemoptysis, prior cancer diagnosis, family history, etc.) and radiologic features (size,</td></tr>
</table>

© 2023 Ganguli I et al. *JAMA Network Open.*

<table>
<tr><td></td><td></td><td>irregular borders, ground glass, new or growing). See PCOI guideline: Incidental Pulmonary Nodules.</td></tr>
<tr><td>Hepatic hemangioma</td><td>Ultrasound, CT, MRI</td><td>Prevalence: Most common benign liver lesion, 0.4-20% in the general population. Hemangiomas are more common in women (ratio 3:1) and in adults 30-50 years old.<br>Prognosis: Most lesions exhibit either slow or no growth and rarely develop complications.<br>Next steps for suspected hemangiomas:<br>Single lesion, low-risk patient: No further imaging<br>Multiple lesions or high-risk patient*: Contrast-enhanced MRI (dual-phase contrast-enhanced CT if MRI is contraindicated)<br>*: high risk patient: cirrhosis, known malignancy known to metastasize to liver, hepatic steatosis, significant alcohol use, liver function abnormalities, hereditary liver disease, hepatitis, anabolic steroid use, choledochal cysts, sclerosing cholangitis<br>Next steps for previously characterized hemangiomas (by MRI or CT):<br>Low risk features and low risk patient: No further imaging.<br>Symptomatic: Surgical consultation, ±contrast-enhanced MRI in 6-12 months.</td></tr>
<tr><td>Renal cyst</td><td>Ultrasound, CT scan</td><td>Prevalence: Simple renal cysts are commonly found in normal kidneys.<br>Rate of incidentally found simple renal cysts on ultrasound:
<table>
<tr><td>Age, y</td><td>Male, %</td><td>Female, %</td></tr>
<tr><td>15-29</td><td>0</td><td>0</td></tr>
<tr><td>30-49</td><td>1.9</td><td>1.4</td></tr>
<tr><td>50-69</td><td>15</td><td>7</td></tr>
<tr><td>>70</td><td>32</td><td>15</td></tr>
</table>
Symptoms: Rarely, hypertension or pain.<br>Prognosis: No risk of malignancy for simple cysts.<br>Next steps:<br>If ultrasound is equivocal, if nodular calcifications or septae are present, or if multiple renal cysts are clustered, renal protocol CT or MRI.<br>If CT or MRI are indeterminate, follow-up CT or MRI in 6-12 months<br>If CT or MRI are suspicious, specialist referral.<br>Complications: Infection, hypertension, hemorrhage.</td></tr>
<tr><td>Adrenal mass</td><td>Abd CT, Abd MRI</td><td>Prevalence: Overall 4% on abdominal CT. Increases with age from 1% for <30yo to 7% for >70yo.<br>Symptoms: Usually asymptomatic. Symptoms related to excess hormone activity, 11% of adrenal masses are hormone-secreting.<br>Prognosis: Usually benign, < 7.5% of adrenal masses are malignant.<br>Next Steps:<br>Biochemical testing for hypercortisolism, pheochromocytoma, and hyperaldosteronism.<br>For further follow-up, see:<br>• PCOI guideline: Adrenal Incidentaloma<br>• Patient handout: Adrenal Nodule</td></tr>
</table>

## Resources

- Page or message the reading radiologist or pathologist
- E-consult radiology on EPIC

### Massachusetts General Hospital

- MGH Dodd Room for specialty consult (White building, floor 2) for specialist consultation or ED reading room for urgent imaging
- MGH Radiology Portal

### Brigham and Women's Hospital

© 2023 Ganguli I et al. *JAMA Network Open.*

- “Drop in” to a BWH Virtual Reading Room speak to a radiologist Monday-Friday, 8am-5pm.

## References

1. O’Sullivan JW, Muntinga T, Grigg S, Ioannidis JPA. Prevalence and outcomes of incidental imaging findings: Umbrella review. *BMJ*. 2018;361. doi:10.1136/bmj.k2387
2. Britt CJ, Maas AM, Kennedy TA, Hartig GK. Incidental Findings on FDG PET/CT in Head and Neck Cancer. *Otolaryngol - Head Neck Surg (United States)*. 2018;158(3):484-488. doi:10.1177/0194599817742579
3. Morris Z, Whiteley WN, Longstreth WT, et al. Incidental findings on brain magnetic resonance imaging: Systematic review and meta-analysis. *BMJ*. 2009;339(7720):547-550. doi:10.1136/bmj.b3016
4. Brinjikji W, Luetmer PH, Comstock B, et al. Systematic literature review of imaging features of spinal degeneration in asymptomatic populations. *Am J Neuroradiol*. 2015;36(4):811-816. doi:10.3174/ajnr.A4173

*This material was created as part of a study funded by the Robert Wood Johnson Foundation. It was developed using literature review, content expert review, and interviews with BWH primary care physicians.*

© 2023 Ganguli I et al. *JAMA Network Open.*

## eMethods 3. Physician Control Group Email

Subject Upcoming Visits for Visit Preparation Trial

Dear Dr. _____,

Thank you for agreeing to participate in this study. We will share **the following tips with 10 of your patients** to help them prepare for their upcoming visits:

1. Bring your medication(s) for the doctor to review
2. If you have seen another doctor or been to the emergency room since your last visit, bring along information about these visits
3. Write down any questions or concerns you want to share with your doctor

Thank you for your commitment to providing outstanding care.

Sincerely,

Ishani Ganguli, MD MPH

## eMethods 4. Reference Materials for Intervention Group Patients

Patient reference materials were developed through a user-centered design process consisting of multiple rounds of patient interviews and solicitation of reactions to drafted materials (5). The patient materials included a website (https://testinfo.bwh.harvard.edu/) with an embedded video, quiz, and link to a PCOI patient handout entitled “Medical Tests: The Basics.” The PCOI handout is also accessible to all patients through the health system patient portal.

All reference materials emphasize three points: first, that medical tests are only one part of a “doctor’s toolbox” that includes other “tools” like eliciting symptoms and family history, and tests are not essential in all circumstances; second, that medical tests have potential benefits and potential downsides; and third, that patients can ask their doctors questions about tests (e.g., “How will this change what we do next?,” “What else can we do instead of this test?”). The materials were written at a fifth-grade reading level. They were designed to be accessible and to incorporate multiple modes of communication (text, audio, and graphics), following literature-informed best practices for patient-facing educational content.

© 2023 Ganguli I et al. *JAMA Network Open.*

## eMethods 5. Survey Instruments

## Patient pre-study survey

**The following two questions are about your approach to medical care in general.**

**1. Sometimes, medical action is clearly necessary, and sometimes it is clearly NOT necessary. Other times, reasonable people differ in their beliefs about whether medical action is needed. In situations where it's not clear, do you tend to lean toward taking action or do you lean toward waiting and seeing if action is needed? Importantly, there is no "right" way to be.**

○ I strongly lean towards waiting and seeing
○ I lean towards waiting and seeing
○ I somewhat lean towards waiting and seeing
○ I somewhat lean towards taking action
○ I lean towards taking action
○ I strongly lean towards taking action

**2. The role you play in the treatment option you choose is important. The next question will tell us how you would like the treatment decision to be made. Please choose one of the following statements that best describes how you would like the decision to be made:**

○ I prefer to make the final decision about what treatment I will receive
○ I prefer to make the final selection of my treatment after seriously considering my doctor's opinion
○ I prefer that my doctor and I share responsibility for deciding which treatment is best for me
○ I prefer that my doctor makes the final decision about which treatment will be used, but seriously considers my opinion
○ I prefer to leave all decisions regarding my treatment to my doctor

**3. How often do you have someone help you read materials about your health?**

○ All of the time
○ Most of the time
○ Some of the time
○ A little of the time
○ None of the time
○ N/A

**4. With which gender identify do you most identify?**

○ Female
○ Male
○ Transgender Female
○ Transgender Male
○ Gender Variant/Non-Conforming
○ Prefer Not to Answer

**5. Are you of Hispanic, Latino or Spanish origin?**

○ Yes
○ No
○ Prefer not to answer

**6. With which racial group do you identify yourself?**

○ White
○ Black or African American
○ Asian or Pacific Islander
○ Native American or Alaskan native
○ Of mixed racial background

- ○ Some other race
- ○ Prefer not to answer

**7. What is the highest degree or level of education you have completed?**
- ○ Some High School
- ○ High School
- ○ Bachelor's Degree
- ○ Master's Degree
- ○ Ph.D. or higher
- ○ Trade School
- ○ Prefer not to say

## Patient post-study survey

**Please answer these questions about your recent visit with your primary care physician.**

**1. How long have you been seeing your primary care physician?**
- ○ Less than 1 year
- ○ 1 year to less than 3 years
- ○ 3 years to less than 5 years
- ○ 5 years to less than 10 years
- ○ 10 years or more
- ○ Don't know

**2. At your recent visit, which type(s) of medical test(s) did you and your doctor talk about? Select every type of tests that you or your doctor mentioned, even if your doctor did not order the test.**
- ☐ Blood test (e.g., blood count, thyroid test)
- ☐ Urine test (e.g., urinalysis)
- ☐ Imaging test (e.g., x-ray, MRI, CT scan) Electrocardiogram (EKG)
- ☐ Other
- ☐ None

Please describe “Other”: ____________________________

*[Question 3-11 are asked if one or more tests are selected for Question 2]*

**3. At your recent visit, who first raised the topic of ordering medical test(s)?**
- ○ Only I raised the idea of getting one or more medical test(s).
- ○ Only my provider raised the idea of getting one or more medical test(s).
- ○ I raised the idea of getting one or more medical tests, and my provider also raised the idea of getting one or more medical tests.

Please describe “Other”: ______________________________

**4. How would you rate your overall level of satisfaction with how your doctor discussed the medical test(s) with you during your recent visit?**
- ○ Very unsatisfied
- ○ Unsatisfied
- ○ Neither satisfied nor satisfied
- ○ Satisfied
- ○ Very satisfied

© 2023 Ganguli I et al. *JAMA Network Open.*

**The next few questions ask about how you and your doctor talked about the medical test(s) during your recent visit.**

**5. During this visit, how much did you and your health care provider talk about the reasons you might want to have the test(s)?**
- ○ A lot
- ○ Some
- ○ A little
- ○ Not at all

**6. During this visit, how much did you and your doctor talk about the reasons you might NOT want to have the test(s)?**
- ○ A lot
- ○ Some
- ○ A little
- ○ Not at all

**7. During this visit, did your doctor talk about alternatives to the test(s) as something you should seriously consider?**
- ○ Yes
- ○ No

**8. During this visit, did your doctor ask if you wanted to have the test(s)?**
- ○ Yes
- ○ No

**9. During this visit, did your doctor discuss how the test(s) would affect the next steps in your care?**
- ○ Yes
- ○ No

**10. In general, during this visit, did the doctor explain the medical test(s) in a way that was easy to understand?**
- ○ Yes, completely
- ○ Yes, a little
- ○ No
- ○ Not applicable

**What factors were important to you in the decision to get or not to get the medical test(s)? (Select all that apply)**
- ☐ The desire to learn something about my body or my health
- ☐ The desire to gather information that may suggest ways to improve my health
- ☐ The desire to make sure that I do not have a health problem
- ☐ The cost that I would pay
- ☐ The cost that my insurance company would pay
- ☐ The potential stress, discomfort, or physical injuries from getting the test(s)
- ☐ The potential for abnormal test results which could lead to unnecessary tests and treatments
- ☐ None of the above

Please add any other comments about how your doctor talked with you about medical tests at this visit:

______________________________________

**This set of questions asks about some facts that doctors think are important for patients to know about medical testing. Please do your best to answer each question.**

© 2023 Ganguli I et al. *JAMA Network Open.*

**11. What piece(s) of information might doctors think about when making medical decisions? (Select all that apply)**

- ☐ Your medical history
- ☐ Your symptoms
- ☐ Your physical exam
- ☐ Your medical test results
- ☐ None of the above

**12. Select the true statement(s) about tests. (Select all that apply)**

- ☐ An abnormal test result means something bad for your health
- ☐ Abnormal test results can lead to worries and unnecessary follow-up tests and treatments that do not improve your health
- ☐ Routine yearly tests (such as blood tests, urine tests, or EKGs) are useful in most cases

**13. Which of the following is an example of a false positive result?**

- ○ Your strep throat test shows a negative result, but you DO have strep throat
- ○ Your strep throat test shows a positive result, and you DO have strep throat
- ○ Your strep throat test shows a positive result, but you do NOT have strep throat
- ○ Your strep throat test result is inconclusive

**14. Which of the following is an example of an incidental or unexpected finding?**

- ○ You get a chest x-ray for fever and cough, and the x-ray shows you have a lung infection
- ○ You get an abdominal CT scan for stomach issues and the scan shows a small dot on your lung
- ○ You get a negative COVID-19 test even though you really have COVID-19.

© 2023 Ganguli I et al. *JAMA Network Open.*

## Physician pre-study survey

1. **As clinicians, we usually consider clinical evidence for a particular medical test when deciding to order the test. In addition to the clinical evidence for the test, if you were to see a patient tomorrow, how important would each of the following be in your decision to order a particular medical test?**

| | Not at all important | Rarely important | Somewhat important | Moderately important | Extremely important |
|---|---|---|---|---|---|
| Community Norms | ○ | ○ | ○ | ○ | ○ |
| Total cost of the test | ○ | ○ | ○ | ○ | ○ |
| Patient out-of-pocket cost for the test | ○ | ○ | ○ | ○ | ○ |
| Fear of missing something | ○ | ○ | ○ | ○ | ○ |
| Patient request | ○ | ○ | ○ | ○ | ○ |
| Desire to be as thorough as possible | ○ | ○ | ○ | ○ | ○ |
| Habit | ○ | ○ | ○ | ○ | ○ |

2. **How frequently do you consider your patients' out-of-pocket costs as you make clinical decisions?**

○ Always
○ Most of the time
○ Sometimes
○ Rarely
○ Never

**It is common for screening and diagnostic tests to show false positives or incidental findings, such as lab test abnormalities or lesions on imaging tests that are unrelated to why you ordered the tests (e.g., CT scans showing pulmonary or adrenal nodules, complete blood counts showing slightly elevated mean corpuscular volume, and urinalyses showing red blood cells.)**

3. **When you are considering a laboratory test (e.g., blood and urine tests), how often do you talk with your patients about the possibility that the test may show false positives or incidental findings?**

○ Always
○ Most of the time
○ Sometimes
○ Rarely
○ Never

4. **When you are considering an imaging test, how often do you talk with your patients about the possibility that the test may show false positives or incidental findings?**

○ Always
○ Most of the time
○ Sometimes
○ Rarely
○ Never

5. **False positives or incidental findings may prompt "cascades" of downstream care such as telephone calls, office visits, further testing, treatments, emergency department visits, hospitalizations, or new diagnoses. Have you ever experienced a cascade for your patients due to a false positive or incidental finding?**

© 2023 Ganguli I et al. *JAMA Network Open.*

- ○ Yes
- ○ No

**6. In the past year, how often did you experience such cascades for your patients?**

- ○ Never
- ○ Once
- ○ A few times in the year
- ○ About once a month
- ○ About once a week
- ○ Several times each week
- ○ Every day

**7. When you are considering ordering a test, how often do you talk with your patient about the possibility that the test may show false positives or incidental findings that may lead to a cascade?**

- ○ Always
- ○ Most of the time
- ○ Sometimes
- ○ Rarely
- ○ Never

**7a. How do you explain the concept of "cascades" to your patients? (this question is optional)**

________________________

**8. What are barriers to discussing the possibility of cascades with your patients when considering a test? Check all that apply.**

- ☐ I do not consider cascades to be worth a discussion
- ☐ I am not sure what to say about possible cascades
- ☐ I am not comfortable discussing cascades
- ☐ I am concerned it will lead to distrust
- ☐ I am concerned it will confuse my patients
- ☐ I don't have enough time
- ☐ Other
- ☐ None

Please describe “Other”: ______________________________

**9. What year did you finish residency? _____________________**

**10. With which gender identify do you most identify?**

- ○ Female
- ○ Male
- ○ Transgender Female
- ○ Transgender Male
- ○ Gender Variant/Non-Conforming
- ○ Prefer Not to Answer

**11. Are you of Hispanic, Latino or Spanish origin?**

- ○ Yes
- ○ No
- ○ Prefer not to answer

© 2023 Ganguli I et al. *JAMA Network Open.*

**12. With which racial group do you identify yourself?**

- ○ White
- ○ Black or African American
- ○ Asian or Pacific Islander
- ○ Native American or Alaskan native
- ○ Of mixed racial background
- ○ Some other race
- ○ Prefer not to answer

**13. Approximately what percentage of your professional time is spent in direct outpatient primary care?**

- ○ < 25%
- ○ 25-49%
- ○ 50-74%
- ○ > 75%

## Physician post-study survey

**1. As clinicians, we usually consider clinical evidence for a particular medical test when deciding to order the test. In addition to the clinical evidence for the test, if you were to see a patient tomorrow, how important would each of the following be in your decision to order a particular medical test?**

| | Not at all important | Rarely important | Somewhat important | Moderately important | Extremely important |
|---|---|---|---|---|---|
| Community Norms | ○ | ○ | ○ | ○ | ○ |
| Total cost of the test | ○ | ○ | ○ | ○ | ○ |
| Patient out-of-pocket cost for the test | ○ | ○ | ○ | ○ | ○ |
| Fear of missing something | ○ | ○ | ○ | ○ | ○ |
| Patient request | ○ | ○ | ○ | ○ | ○ |
| Desire to be as thorough as possible | ○ | ○ | ○ | ○ | ○ |
| Habit | ○ | ○ | ○ | ○ | ○ |

**2. How frequently do you consider your patients' out-of-pocket costs as you make clinical decisions?**

- ○ Always
- ○ Most of the time
- ○ Sometimes
- ○ Rarely
- ○ Never

© 2023 Ganguli I et al. *JAMA Network Open.*

**It is common for screening and diagnostic tests to show false positives or incidental findings, such as lab test abnormalities or lesions on imaging tests that are unrelated to why you ordered the tests (e.g., CT scans showing pulmonary or adrenal nodules, complete blood counts showing slightly elevated mean corpuscular volume, and urinalyses showing red blood cells.)**

**3. When you are considering a laboratory test (e.g., blood and urine tests), how often do you talk with your patients about the possibility that the test may show false positives or incidental findings?**

- ○ Always
- ○ Most of the time
- ○ Sometimes
- ○ Rarely
- ○ Never

**4. When you are considering an imaging test, how often do you talk with your patients about the possibility that the test may show false positives or incidental findings?**

- ○ Always
- ○ Most of the time
- ○ Sometimes
- ○ Rarely
- ○ Never

**5. False positives or incidental findings may prompt “cascades” of downstream care such as telephone calls, office visits, further testing, treatments, emergency department visits, hospitalizations, or new diagnoses. When you are considering ordering a test, how often do you talk with your patient about the possibility that the test may show false positives or incidental findings that may lead to a cascade?**

- ○ Always
- ○ Most of the time
- ○ Sometimes
- ○ Rarely
- ○ Never

**5a. How do you explain the concept of "cascades" to your patients? (this question is optional)**

________________________

**6. What are barriers to discussing the possibility of cascades with your patients when considering a test? Check all that apply.**

- ☐ I do not consider cascades to be worth a discussion
- ☐ I am not sure what to say about possible cascades
- ☐ I am not comfortable discussing cascades
- ☐ I am concerned it will lead to distrust
- ☐ I am concerned it will confuse my patients
- ☐ I don't have enough time
- ☐ Other
- ☐ None

Please describe “Other”: _______________________________

© 2023 Ganguli I et al. *JAMA Network Open.*

## eMethods 6. Exit Interview Guides

**Patient questions:**

1. General experience with the intervention
    a. Prior to the study, tell me about what kind of thoughts or feelings you had about medical tests. Did you have any concerns about them, such as costs or time?
    b. Did you receive the email or text message before medical visit with some educational materials about talking with your doctor about medical tests?
        i. What did you think of it?
        ii. What do you remember from it?
2. Visit
    a. Tell me about your visit with your primary care doctor. What happened during that visit? Was it different in any way compared to usual?
    b. Did you discuss medical tests at all? How did that conversation go?
        i. Did you raise the topic? If so, why?
    c. Did you ask any questions about medical tests?
        i. Why? Or why not?
        ii. Did you feel comfortable asking about testing?
3. Overall
    a. What do you remember learning from the educational materials?
    b. Other than medical tests, what kinds of things can your doctor do to understand your health?
    c. How would you describe the reasons to get or not get medical tests?
    d. Is there anything else you'd like to share with us?

**Physician questions:**

1. General Experience with Intervention
    a. Prior to the study, what types of medical tests did you routinely order during an annual physical, say for a healthy 40-year-old woman?
    b. Did you receive the study email we sent about a week before your first study patient's physical exam?
        i. What did you think of it?
        ii. How did you react/feel when you read it?
        iii. Were you surprised by how you compared to other BWH physicians?
        iv. Tell us about your usual test ordering habits and reasoning?
    c. Did you review any of the additional resources attached to/linked in the study email (e.g. medical test interpretation PCOI, incidental findings PCOI, external links)?
        i. What did you think of these resources?
    d. Did you review the patient website/PCOI handout that we linked/attached to this email on the basics of medical tests?
        i. What did you think of it?
        ii. How thoroughly did you review these materials? How much time did you spend with them?
2. Impact of Intervention
    a. Did these emails and resources influence your thinking during patient visits? How so?
    b. How, if at all, did these materials change conversations with patients about medical tests?
        i. Did patients bring up the materials they were sent?
        ii. Did you bring up the materials with the patients?
        iii. Did you talk about medical tests differently than you might have before?
            1. Why or why not?
            2. Examples?
    c. Will this change anything about the way you will discuss medical tests with patients going forward?

© 2023 Ganguli I et al. *JAMA Network Open.*

i. Why or why not?
ii. Which types of medical tests might it impact?

d. How do you assess whether getting a medical test is important to a patient? Does this influence your test ordering decisions?

3. Wrap Up
   a. Do you have any additional thoughts you'd like to share with us?

© 2023 Ganguli I et al. *JAMA Network Open.*

## eTable 1. Baseline Patient Characteristics Stratified by Final Survey Completion Status

| Patient Characteristics | | All patients | | | Patients who finished post-study survey | | | Patients who did not finish post-study survey | | |
|---|---|---|---|---|---|---|---|---|---|---|
| | | **Intervention group (n = 166)** | **Control group (n = 148)** | ***p*** | **Intervention group (n = 124)** | **Control group (n = 124)** | ***p*** | **Intervention group (n = 42)** | **Control group (n = 24)** | ***p*** |
| Age at time of visit, mean (SD) | | 50.6 (15.8) | 49.8 (14.8) | 0.69 | 50.8 (15.3) | 51.1 (14.6) | 0.89 | 50.0 (17.3) | 42.9 (14.3) | 0.19 |
| Gender, No. (%) | Male | 57 (34.3) | 43 (29.1) | 0.74 | 44 (35.5) | 38 (30.6) | 0.52 | 13 (31) | 5 (20.8) | 0.25 |
| | Female | 107 (64.5) | 103 (69.6) | | 78 (62.9) | 86 (69.4) | | 29 (69) | 17 (70.8) | |
| | Other* | 2 (1.2) | 2 (1.4) | | 2 (1.6) | 0 (0) | | 0 (0) | 2 (8.3) | |
| Race and ethnicity, No. (%) | Non-Hispanic White | 126 (75.9) | 120 (81.1) | 0.59 | 93 (75.0) | 104 (83.9) | 0.52 | 33 (78.6) | 16 (66.7) | 0.49 |
| | Non-Hispanic Black | 6 (3.6) | 4 (2.7) | | 5 (4.0) | 2 (1.6) | | 1 (2.4) | 2 (8.3) | |
| | Hispanic | 10 (6.0) | 3 (2.0) | | 8 (6.5) | 1 (0.8) | | 2 (4.8) | 2 (8.3) | |
| | Asian/Pacific Islander | 15 (9.0) | 10 (6.8) | | 11 (8.9) | 10 (8.1) | | 4 (9.5) | 0 (0.0) | |
| | Other† | 9 (5.4) | 11 (7.4) | | 7 (5.6) | 7 (5.6) | | 2 (4.8) | 4 (16.7) | |
| Highest educational attainment, No. (%) | No bachelor's degree | 12 (7.2) | 27 (18.2) | 0.07 | 9 (7.3) | 22 (17.7) | 0.04 | 3 (7.1) | 5 (20.8) | 0.45 |
| | Bachelor's degree | 68 (41.0) | 50 (33.8) | | 51 (41.1) | 41 (33.1) | | 17 (40.5) | 9 (37.5) | |
| | Post-graduate degree | 85 (51.2) | 71 (48.0) | | 63 (50.8) | 61 (49.2) | | 22 (52.4) | 10 (41.7) | |
| | Prefer not to say | 1 (0.6) | 0 (0.0) | | 1 (0.8) | 0 (0.0) | | 0 (0) | 0 (0) | |
| Primary insurance, No. (%) | Commercial | 142 (85.5) | 120 (81.1) | 0.79 | 108 (87.1) | 103 (83.1) | 0.87 | 34 (81.0) | 17 (70.8) | 0.46 |
| | Medicaid | 8 (4.8) | 11 (7.4) | | 5 (4.0) | 6 (4.8) | | 3 (7.1) | 5 (20.8) | |
| | Medicare | 14 (8.4) | 12 (8.1) | | 10 (8.1) | 12 (9.7) | | 4 (9.5) | 0 (0.0) | |
| | Other‡ | 2 (1.2) | 5 (3.4) | | 1 (0.8) | 3 (2.4) | | 1 (2.4) | 2 (8.3) | |
| Less than 3 years with current PCP§, No. (%) | | 55 (17.5) | 83 (26.3) | 0.13 | 37 (11.8) | 70 (22.3) | 0.10 | 18 (5.7) | 13 (4.0) | 0.52 |
| Prefers to wait and see (vs. take action, on medical decisions), No. (%) | | 103 (62.0) | 88 (59.5) | 0.62 | 77 (62.1) | 73 (58.9) | 0.60 | 26 (61.9) | 15 (62.5) | 0.95 |
| Prefers patient makes final medical decision (vs. doctor or both), No. (%) | | 72 (43.4) | 61 (41.2) | 0.73 | 53 (42.7) | 50 (40.3) | 0.74 | 19 (45.2) | 11 (45.8) | 0.94 |
| How often someone helps read health materials, No. (%) | All or some of the time | 25 (15.1) | 24 (16.2) | 0.90 | 22 (17.7) | 21 (16.9) | 0.87 | 3 (7.1) | 3 (12.5) | 0.82 |
| | A little of the time | 42 (25.3) | 36 (24.3) | | 33 (26.6) | 31 (25.0) | | 9 (21.4) | 5 (20.8) | |
| | None of the time | 95 (57.2) | 85 (57.4) | | 66 (53.2) | 70 (56.5) | | 29 (69.0) | 15 (62.5) | |
| | Not applicable | 4 (2.4) | 3 (2.0) | | 3 (2.4) | 2 (1.6) | | 1 (2.4) | 1 (4.2) | |

Abbreviations: PCP, primary care physician; SD, standard deviation. p values were calculated using linear mixed effect modeling for continuous variable (age) and chi-square tests with Rao-Scott adjustment for other variables; standard errors were clustered by matched pair and by physician nested within matched pair.

* Other includes gender variant/non-conforming and prefer not to answer.

† Other includes non-Hispanic of mixed racial background, some other race, and prefer not to answer.

‡ Other includes uninsured, worker's compensation, and UniCare GIC. § Years with PCP includes multiply-imputed values for 74 patients.

© 2023 Ganguli I et al. *JAMA Network Open.*

## eTable 2. Comparison of Patient-Reported Test Conversation Measures by Treatment Group

| Patient outcomes | Unadjusted | | | Adjusted for baseline covariates | | |
|---|---|---|---|---|---|---|
| | Intervention group (n = 166) | Control group (n = 148) | Attributable Risk (95% CI) | Intervention group (n = 163)* | Control group (n = 146)* | Attributable Risk (95% CI) |
| SDMP_4, mean (SD) [†, ‡] | 2.12 (1.56) | 1.97 (1.52) | 0.15 (-0.22, 0.52) | 2.11 (1.61) | 1.97 (1.55) | 0.14 (-0.25, 0.54) |
| Medical testing knowledge, mean (SD) [§] | 2.73 (0.97) | 2.55 (0.90) | 0.18 (-0.06, 0.42) | 2.74 (0.98) | 2.54 (0.91) | 0.19 (-0.05, 0.43) |
| Presence of a test conversation, No. (%) | 158 (95.1) | 145 (98.0) | -2.9 (-7.2, 1.4) | 155 (95.4) | 144 (98.3) | -2.9 (-7.0, 1.2) |
| Satisfaction with testing, No. (%)[†] | 113 (71.3) | 95 (65.8) | 5.6 (-5.6, 16.8) | 109 (70.7) | 94 (65.6) | 5.1 (-6.5, 16.7) |
| Discussion about next steps, No. (%)[†] | 110 (69.5) | 103 (71.1) | -1.6 (-12.7, 9.4) | 108 (69.8) | 102 (71.4) | -1.7 (-12.8, 9.5) |
| Doctor explained tests in way that was easy to understand, No. (%)[†] | 132 (83.5) | 126 (86.8) | -3.2 (-12.8, 6.4) | 130 (83.7) | 124 (86.8) | -3.1 (-12.7, 6.6) |

Abbreviation: SDMP_4, Shared Decision-Making Process Survey
* Patients were excluded from adjusted analyses if they were missing one or more demographic covariates
[†] These items were only asked if patient indicated tests were discussed during the study visit. For unadjusted analyses: intervention group: n = 158, control group: n = 145; for adjusted analyses: intervention group: n = 155, control group n = 143.
[‡] Scale from 0-4, in which higher values indicate more components discussed
[§] Scale from 0-4, in which higher values indicate more medical test questions answered correctly

© 2023 Ganguli I et al. *JAMA Network Open.*

## eTable 3. Patient Responses to Individual Components of SDMP and Medical Test Knowledge Scores by Treatment Group

| **Components of SDMP_4 score:** | **Intervention, No. (%)** | **Control, No. (%)** | **Difference (%)** |
|---|---|---|---|
| 1. Doctor discussed reasons to have tests | 144 (86.6) | 126 (85.1) | 1.5 |
| 2. Doctor discussed reasons to NOT have tests | 47 (28.2) | 37 (25.0) | 3.2 |
| 3. Doctor discussed alternatives to tests | 36 (21.6) | 25 (17.0) | 4.7 |
| 4. Doctor asked whether patient wanted tests | 126 (76.1) | 107 (72.5) | 3.6 |
| **Components of medical test knowledge score:** | **Intervention, No. (%)** | **Control, No. (%)** | **Difference (%)** |
| 1. Selected all pieces of information doctors consider in making medical decisions | 145 (87.2) | 124 (83.6) | 3.6 |
| 2. Chose the true statements about tests from a list* | 16 (9.7) | 1 (0.4) | 9.3 |
| 3. Identified example of false positive | 151 (91.0) | 128 (86.2) | 4.9 |
| 4. Identified example of incidental finding | 143 (86.0) | 125 (84.6) | 1.4 |

Abbreviation: SDMP_4, Shared Decision-Making Process Survey

* The options were: an abnormal test means something bad for your health [correct answer: false], abnormal test results can lead to worries and unnecessary follow-up tests and treatments that do no improve your health [correct answer: true], routine yearly tests (such as blood tests, urine tests, or EKGs) are useful in most cases [correct answer: false]. Participants needed to answer all 3 of these correctly.

## eTable 4. Comparison of Patient-Reported Visit Descriptions and Factors Important to Testing Decisions by Treatment Group

| Patient exploratory outcomes | | Unadjusted | | | Adjusted for baseline covariates | | |
|---|---|---|---|---|---|---|---|
| | | Intervention group (n = 166) | Control group (n = 148) | Attributable Risk (95% CI) | Intervention group (n = 163)* | Control group (n = 146)* | Attributable Risk (95% CI) |
| Tests discussed, (%) | Blood test | 145 (87.2) | 137 (92.3) | -5.1 (-13.4, 3.2) | 144 (88.1) | 135 (92.8) | -4.6 (-12.8, 3.5) |
| | Urine test | 53 (31.6) | 22 (14.6) | 17.0 (7.0, 27.1) | 51 (31.5) | 21 (14.5) | 17.1 (7.2, 27.0) |
| | Imaging test | 47 (28.2) | 46 (31.3) | -3.0 (-14.4, 8.3) | 44 (27.2) | 44 (30.0) | -2.9 (-13.9, 8.1) |
| | EKG | 13 (8.1) | 13 (8.8) | -0.7 (-7.2, 5.8) | 13 (8.1) | 12 (8.5) | -0.3 (-6.9, 6.2) |
| | Other | 29 (17.5) | 34 (23.3) | -5.7 (-15.6, 4.1) | 27 (16.6) | 32 (22.2) | -5.6 (-15.5, 4.3) |
| | None | 8 (4.9) | 3 (2.0) | 2.9 (-1.4, 7.2) | 8 (4.6) | 2 (1.7) | 2.9 (-1.2, 7.0) |
| Who raised the idea of ordering test? No. (%)† | Only patient | 8 (6.9) | 4 (3.3) | 3.6 (-1.8, 9.1) | 5 (4.7) | 3 (2.4) | 2.3 (-2.0, 6.5) |
| | Only doctor | 47 (40.5) | 47 (38.5) | 2.0 (-10.6, 14.6) | 46 (40.0) | 47 (38.2) | 1.8 (-10.8, 14.4) |
| | Both | 56 (48.3) | 66 (54.1) | -5.8 (-17.8, 6.1) | 55 (48.3) | 66 (54.0) | -5.7 (-17.8, 6.4) |
| | Other | 5 (4.3) | 5 (4.1) | 0.2 (-5.0, 5.5) | 5 (4.0) | 4 (3.6) | 0.4 (-4.4, 5.2) |
| Factors important to testing decisions, No. (%)† | Learn something about body/health | 102 (64.8) | 77 (53.3) | 11.5 (-0.6, 23.6) | 102 (66.0) | 77 (54.1) | 11.9 (-0.2, 24.0) |
| | Gather information about improving health | 88 (55.9) | 84 (58.0) | -2.0 (-13.9, 9.8) | 87 (56.3) | 83 (57.9) | -1.6 (-13.6, 10.4) |
| | Rule out a health problem | 119 (75.2) | 110 (75.9) | -0.7 (-11.3, 9.9) | 117 (75.6) | 108 (75.8) | -0.3 (-11.0, 10.5) |
| | Out of pocket cost | 14 (8.7) | 9 (5.9) | 2.8 (-3.6, 9.1) | 13 (8.1) | 8 (5.6) | 2.5 (-3.6, 8.6) |
| | Cost to insurance company | 7 (4.3) | 7 (5.0) | -0.6 (-6.0, 4.8) | 6 (4.1) | 7 (4.7) | -0.7 (-5.6, 4.2) |
| | Potential stress, discomfort, or physical injury from test | 17 (11.1) | 12 (8.5) | 2.5 (-4.6, 9.7) | 16 (10.4) | 11 (7.9) | 2.4 (-4.5, 9.4) |
| | Potential for abnormal results that could lead to unnecessary tests/treatments | 11 (7.1) | 7 (4.9) | 2.2 (-3.8, 8.2) | 10 (6.8) | 7 (5.0) | 1.7 (-4.2, 7.7) |
| | None of the above | 10 (6.4) | 6 (4.0) | 2.4 (-3.1, 7.9) | 9 (6.1) | 5 (3.7) | 2.4 (-2.7, 7.5) |

***** Patients were excluded from adjusted analyses if they were missing one or more demographic covariates

† Questions were only asked if patient indicated tests were discussed during the study visit. For unadjusted analyses: intervention group: n = 158, control group: n = 145; for adjusted analyses: intervention group: n = 155, control group n = 143.

© 2023 Ganguli I et al. *JAMA Network Open.*

## eTable 5. Summary of Physician Test Ordering Preferences and Behaviors, Prestudy and Poststudy, by Treatment Group

| Physician exploratory outcomes | | Intervention group (n = 10) | | | Control group (n = 10) | | |
|---|---|---|---|---|---|---|---|
| | | Pre-study survey | Post-study survey | Difference (pre – post) | Pre-study survey | Post-study survey | Difference (pre – post) |
| When ordering tests, how important is/are: | | | | | | | |
| Community norms, No. (%) | Not at all/rarely | 3 (30) | 2 (20) | -1 (-10) | 5 (50) | 1 (10) | -4 (-40) |
| | Somewhat | 4 (40) | 6 (60) | 2 (20) | 2 (20) | 4 (40) | 2 (20) |
| | Moderately/extremely | 3 (30) | 2 (20) | -1 (-10) | 3 (30) | 5 (50) | 2 (20) |
| Total cost of test, No. (%) | Not at all/rarely | 1 (10) | 1 (10) | 0 (0) | 2 (20) | 0 (0) | -2 (-20) |
| | Somewhat | 7 (70) | 7 (70) | 0 (0) | 3 (30) | 5 (50) | 2 (20) |
| | Moderately/extremely | 2 (20) | 2 (20) | 0 (0) | 5 (50) | 5 (50) | 0 (0) |
| Patient out-of-pocket cost for test, No. (%) | Not at all/rarely | 0 (0) | 1 (10) | 1 (10) | 0 (0) | 1 (10) | 1 (10) |
| | Somewhat | 4 (40) | 2 (20) | -2 (-20) | 2 (20) | 1 (10) | -1 (-10) |
| | Moderately/extremely | 6 (60) | 7 (70) | 1 (10) | 8 (80) | 8 (80) | 0 (0) |
| Fear of missing something, No. (%) | Not at all/rarely | 1 (10) | 3 (30) | 2 (20) | 3 (30) | 2 (20) | -1 (-10) |
| | Somewhat | 3 (30) | 3 (30) | 0 (0) | 2 (20) | 2 (20) | 0 (0) |
| | Moderately/extremely | 6 (60) | 4 (40) | -2 (-20) | 5 (50) | 6 (60) | 1 (10) |
| Patient request, No. (%) | Not at all/rarely | 1 (10) | 0 (0) | -1 (-10) | 1 (10) | 0 (0) | -1 (-10) |
| | Somewhat | 6 (60) | 8 (80) | 2 (20) | 4 (40) | 6 (60) | 2 (20) |
| | Moderately/extremely | 3 (30) | 2 (20) | -1 (-10) | 5 (50) | 4 (40) | -1 (-10) |
| Desire to be as thorough as possible, No. (%) | Not at all/rarely | 1 (10) | 1 (10) | 0 (0) | 1 (10) | 0 (0) | -1 (-10) |
| | Somewhat | 4 (40) | 4 (40) | 0 (0) | 3 (30) | 3 (30) | 0 (0) |
| | Moderately/extremely | 5 (50) | 5 (50) | 0 (0) | 6 (60) | 7 (70) | 1 (10) |
| Habit, No. (%) | Not at all/rarely | 6 (60) | 6 (60) | 0 (0) | 5 (50) | 2 (20) | -3 (-30) |
| | Somewhat | 3 (30) | 0 (0) | -3 (-30) | 3 (30) | 8 (80) | 5 (50) |
| | Moderately/extremely | 1 (10) | 4 (40) | 3 (30) | 2 (20) | 0 (0) | -2 (-20) |
| How frequently do you consider your patient's out-of-pocket costs in clinical decision? No. (%) | Rarely/never | 2 (20) | 1 (10) | -1 (-10) | 0 (0) | 0 (0) | 0 (0) |
| | Sometimes | 1 (10) | 4 (40) | 3 (30) | 4 (40) | 5 (50) | 1 (10) |
| | Always/most of the time | 7 (70) | 5 (50) | -2 (-20) | 6 (60) | 5 (50) | -1 (-10) |
| Before laboratory test: how often do you discuss the possibility of false positive/incidental findings? No. (%) | Rarely/never | 4 (40) | 5 (50) | 1 (10) | 5 (50) | 4 (40) | -1 (-10) |
| | Sometimes | 6 (60) | 4 (40) | -2 (-20) | 3 (30) | 5 (50) | 2 (20) |
| | Always/most of the time | 0 (0) | 1 (10) | 1 (10) | 2 (20) | 1 (10) | -1 (-10) |

© 2023 Ganguli I et al. *JAMA Network Open.*

## eTable 5. Summary of Physician Test Ordering Preferences and Behaviors, Prestudy and Poststudy, by Treatment Group (continued)

| Physician exploratory outcomes | | Intervention Group (n = 10) | | | Control Group (n = 10) | | |
|---|---|---|---|---|---|---|---|
| | | Pre-study survey | Post-study survey | Difference (pre – post) | Pre-study survey | Post-study survey | Difference (pre – post) |
| Before imaging test: how often do you discuss the possibility of false positive/incidental findings? No. (%) | Rarely/never | 1 (10) | 0 (0) | -1 (-10) | 1 (10) | 1 (10) | 0 (0) |
| | Sometimes | 7 (70) | 8 (80) | 1 (10) | 6 (60) | 8 (80) | 2 (20) |
| | Always/most of the time | 2 (20) | 2 (20) | 0 (0) | 3 (30) | 1 (10) | -2 (-20) |
| Before any test, frequency of discussing possibility of cascades? No. (%) | Rarely/never | 3 (30) | 2 (20) | -1 (-10) | 5 (50) | 2 (20) | -3 (-30) |
| | Sometimes | 6 (60) | 7 (70) | 1 (10) | 5 (50) | 8 (80) | 3 (30) |
| | Always/most of the time | 1 (10) | 1 (10) | 0 (0) | 0 (0) | 0 (0) | 0 (0) |
| Barriers to discussing possibility of cascades, No. (%) | Not worth a discussion | 0 (0) | 1 (10) | 1 (10) | 0 (0) | 0 (0) | 0 (0) |
| | Not sure what to say | 0 (0) | 0 (0) | 0 (0) | 2 (20) | 0 (0) | -2 (-20) |
| | Not comfortable discussing cascades | 0 (0) | 0 (0) | 0 (0) | 0 (0) | 0 (0) | 0 (0) |
| | Concerned will lead to distrust | 1 (10) | 1 (10) | 0 (0) | 1 (10) | 0 (0) | -1 (-10) |
| | Concerned about confusing patients | 3 (30) | 6 (60) | 3 (30) | 7 (70) | 8 (80) | 1 (10) |
| | Don’t have enough time | 8 (80) | 7 (70) | -1 (-10) | 9 (90) | 7 (70) | -2 (-20) |
| | Other | 0 (0) | 0 (0) | 0 (0) | 1 (10) | 1 (10) | 0 (0) |

There was one missing physician post-study survey, results were imputed using multiple imputation.

© 2023 Ganguli I et al. *JAMA Network Open.*

## eTable 6. Patient Analyses Stratified by Physician Gender and Patient Years With PCP

| Stratified by physician gender | Male physician | | | Female physician | | |
|---|---|---|---|---|---|---|
| | **Intervention (n = 62)** | **Control (n = 44)** | **Attributable Risk (95% CI)** | **Intervention (n = 104)** | **Control (n = 104)** | **Attributable Risk (95% CI)** |
| SDMP_4, mean (SD)* | 1.96 (1.56) | 1.83 (1.66) | 0.13 (-1.04, 1.30) | 2.22 (1.65) | 2.02 (1.54) | 0.19 (-0.36, 0.74) |
| Medical test knowledge, mean (SD) | 2.65 (0.94) | 2.62 (1.01) | 0.03 (-0.67, 0.72) | 2.78 (1.04) | 2.52 (0.93) | 0.26 (-0.07, 0.59) |
| Satisfaction with testing, No. (%)* | 39 (67.4) | 33 (75.6) | -8.2 (-28.6, 12.2) | 74 (73.7) | 62 (61.5) | 12.2 (-1.0, 25.3) |
| Discussion about next steps, No. (%)* | 43 (73.3) | 36 (81.2) | -7.9 (-24.4, 8.6) | 67 (67.3) | 67 (66.8) | 0.5 (-13.8, 14.9) |
| Doctor explained tests in way that was easy to understand, No. (%)* | 47 (80.2) | 40 (90.5) | -10.3 (-25.7, 5.1) | 85 (85.6) | 86 (85.2) | 0.4 (-10.9, 11.6) |
| Discussed test(s) during visit, No. (%) | 58 (93.5) | 44 (100.0) | -6.5 (-13.0, 0.1) | 100 (96.0) | 101 (97.1) | -1.1 (-6.7, 4.4) |
| **Stratified by years with PCP** | **3 years or more** | | | **Less than 3 years** | | |
| | **Intervention (n = 55)** | **Control (n = 82)** | **Attributable Risk (95% CI)** | **Intervention (n = 111)** | **Control (n = 66)** | **Attributable Risk (95% CI)** |
| SDMP_4, mean (SD)* | 2.03 (1.47) | 2.01 (1.40) | 0.02 (-0.61, 0.65) | 2.12 (1.72) | 1.93 (1.50) | 0.19 (-0.37, 0.74) |
| Medical test knowledge, mean (SD) | 2.64 (1.13) | 2.46 (1.05) | 0.18 (-0.30, 0.65) | 2.75 (0.98) | 2.66 (0.90) | 0.09 (-0.24, 0.42) |
| Satisfaction with testing, No. (%)* | 35 (68.0) | 45 (56.2) | 11.8 (-5.2, 28.7) | 78 (72.9) | 50 (77.7) | -4.8 (-19.6, 10.0) |
| Discussion about next steps, No. (%)* | 36 (71.4) | 56 (69.2) | 2.2 (-15.9, 20.4) | 73 (68.6) | 47 (73.6) | -5.0 (-19.8, 9.7) |
| Doctor explained tests in way that was easy to understand, No. (%)* | 40 (78.8) | 68 (84.9) | -6.1 (-22.7, 10.4) | 92 (85.9) | 57 (89.0) | -3.1 (-14.4, 8.2) |
| Discussed test(s) during visit, No. (%) | 51 (92.4) | 81 (97.6) | -5.2 (-13.1, 2.7) | 107 (96.4) | 64 (98.5) | -2.1 (-6.5, 2.4) |

Abbreviation: SDMP_4, Shared Decision-Making Process Survey
* Questions were only asked if patient indicated tests were discussed during the study visit.
These analyses were not adjusted for covariates due to computational barriers in the setting of small sample sizes.

Among patients with female PCPs, those in the intervention group reported higher satisfaction than those in the control group, while the reverse was observed for patients with male PCPs, although differences were not statistically significant (female physicians: 73.7% intervention vs. 61.5% control, difference(95%CI) 12.2% (-1.0%, 25.3%); male physicians: 67.4% intervention, 75.6% control, difference(95%CI) -8.2% (-28.6%, 12.2%)). In addition, patients who had been with their PCP for less than 3 years reported higher satisfaction with the testing discussion in the intervention group than the control group, with the reverse observed among patients who had been with their PCP for 3 years or more (<3 years: 68% intervention vs 56.2% control, difference(95%CI) 11.8% (-5.2%, 28.7%); >=3 years: 72.9% intervention, 77.7% control, difference(95%CI) -4.7% (-19.6%-10.0%)). These differences were not statistically significant.

© 2023 Ganguli I et al. *JAMA Network Open.*

## eTable 7. Patient Analyses Stratified by Patient Preferences About Medical Decision-Making

| Stratified by approach to medical decision-making | Prefer to take action | | | Prefer to wait and see | | |
|---|---|---|---|---|---|---|
| | Intervention (n = 63) | Control (n = 60) | Attributable Risk (95% CI) | Intervention (n = 103) | Control (n = 88) | Attributable Risk (95% CI) |
| SDMP_4, mean (SD)* | 2.09 (1.48) | 2.01 (1.41) | 0.08 (-0.43, 0.59) | 2.13 (1.53) | 1.96 (1.46) | 0.17 (-0.21, 0.55) |
| Medical test knowledge, mean (SD) | 2.78 (0.87) | 2.62 (0.77) | 0.16 (-0.19, 0.50) | 2.69 (0.94) | 2.50 (0.88) | 0.19 (-0.07, 0.45) |
| Satisfaction with testing, No. (%)* | 47 (75.6) | 36 (62.4) | 13.2 (-5.1, 31.5) | 66 (68.7) | 59 (68.0) | 0.6 (-13.7, 15.0) |
| Discussion about next steps, No. (%)* | 44 (71.6) | 40 (68.4) | 3.2 (-16.2, 22.6) | 65 (68.2) | 64 (73.0) | -4.9 (-19.7, 10.0) |
| Doctor explained tests in way that was easy to understand, No. (%)* | 50 (80.0) | 50 (85.4) | -5.4 (-21.3, 10.5) | 82 (86.0) | 76 (87.7) | -1.7 (-14.4, 10.9) |
| Discussed test(s) during visit, No. (%) | 62 (98.4) | 58 (96.7) | 1.7 (-3.9, 7.3) | 96 (93.1) | 87 (98.9) | -5.8 (-11.9, 0.2) |
| **Stratified by preference for who makes final medical decision** | **Patient makes decision** | | | **Physician makes decision or decision is shared** | | |
| | **Intervention (n = 72)** | **Control (n = 61)** | **Attributable Risk (95% CI)** | **Intervention (n = 94)** | **Control (n = 87)** | **Attributable Risk (95% CI)** |
| SDMP_4, mean (SD)* | 2.20 (1.40) | 2.09 (1.32) | 0.11 (-0.36, 0.58) | 2.00 (1.53) | 1.93 (1.51) | 0.06 (-0.39, 0.52) |
| Medical test knowledge, mean (SD) | 2.77 (0.91) | 2.56 (0.86) | 0.22 (-0.10, 0.53) | 2.70 (0.97) | 2.55 (0.91) | 0.15 (-0.17, 0.47) |
| Satisfaction with testing, No. (%)* | 47 (68.7) | 36 (61.8) | 6.9 (-10.2, 24.1) | 66 (73.4) | 60 (68.4) | 5 (-9.5, 19.4) |
| Discussion about next steps, No. (%)* | 50 (73.5) | 41 (70.3) | 3.3 (-15.9, 22.5) | 60 (66.5) | 62 (71.8) | -5.2 (-19.5, 9.0) |
| Doctor explained tests in way that was easy to understand, No. (%)* | 61 (89.2) | 49 (84.5) | 4.7 (-8.7, 18.2) | 71 (79.2) | 77 (88.4) | -9.1 (-21.5, 3.2) |
| Discussed test(s) during visit, No. (%) | 68 (94.3) | 58 (95.1) | -0.8 (-8.3, 6.7) | 90 (95.7) | 87 (100.0) | -4.3 (-8.4, -0.2) |

Abbreviation: SDMP_4: Shared Decision-Making Process Survey; SD, standard deviation
* Questions were only asked if patient indicated tests were discussed during the study visit.
These analyses were not adjusted for covariates due to computational barriers in the setting of small sample sizes.

© 2023 Ganguli I et al. *JAMA Network Open.*

## eTable 8. Baseline Characteristics of Interviewed Physicians and Patients

| **Physician Characteristics** | | **All enrolled physicians (n = 20)** | **Intervention group physicians (n = 10)** | **Interviewed physicians (n = 3)** |
|---|---|---|---|---|
| Female sex, No. (%) | | 13 (65) | 6 (60) | 2 (67) |
| Race/ethnicity, No. (%) | Non-Hispanic White | 13 (65) | 7 (70) | 3 (100) |
| | Asian/Pacific Islander | 4 (20) | 2 (20) | 0 (0) |
| | Other* | 3 (15) | 1 (10) | 0 (0) |
| Years since residency completion, mean (SD) | | 19.7 (11.7) | 21.8 (14.5) | 22.0 (11.8) |
| Percentage of professional time spent in outpatient primary care | 25-74% | 6 (30) | 5 (50) | 2 (67) |
| | 75% + | 14 (70) | 5 (50) | 1 (33) |
| Frequency of experiencing cascades of care in clinical work | Once/year to a few times/year | 11 (55) | 5 (50) | 0 (0) |
| | Once/month to once/week | 9 (45) | 5 (50) | 3 (100) |
| **Patient Characteristics** | | **All enrolled patients (n = 314)** | **Intervention group patients (n = 166)** | **Interviewed patients (n = 16)** |
| Age at time of visit, mean (SD) | | 50.2 (15.3) | 50.6 (15.8) | 51.4 (14.2) |
| Sex, No. (%) | Female | 210 (66.9) | 107 (64.5) | 9 (56.3) |
| | Male | 100 (31.8) | 57 (34.3) | 7 (43.8) |
| | Other † | 4 (1.3) | 2 (1.2) | 0 (0.0) |
| Race/ethnicity, No. (%) | Non-Hispanic White | 246 (78.3) | 126 (75.9) | 12 (75.0) |
| | Asian/Pacific Islander | 25 (8.0) | 15 (9.0) | 1 (6.3) |
| | Non-Hispanic Black | 10 (3.2) | 6 (3.6) | 1 (6.3) |
| | Hispanic | 13 (4.1) | 10 (6.0) | 1 (6.3) |
| | Other* | 20 (6.4) | 9 (5.4) | 1 (6.3) |
| Highest educational attainment | No bachelor’s degree | 39 (12.4) | 12 (7.2) | 0 (0.0) |
| | Bachelor’s degree | 118 (37.6) | 68 (41.0) | 7 (43.8) |
| | Post-graduate degree | 156 (49.7) | 85 (51.2) | 9 (56.3) |
| | Prefer not to answer | 1 (0.3) | 1 (0.6) | 0 (0.0) |
| Primary insurance, No. (%) | Commercial | 262 (83.4) | 142 (85.5) | 14 (87.5) |
| | Medicaid | 19 (6.1) | 8 (4.8) | 2 (12.5) |
| | Medicare | 26 (8.3) | 14 (8.4) | 0 (0.0) |
| | Other ‡ | 7 (2.2) | 2 (1.2) | 0 (0.0) |
| Less than 3 years with current PCP§, No. (%) | | 138 (43.8) | 55 (17.5) | 4 (25.0) |
| Prefers to wait and see (vs. take action) on medical decisions, No. (%) | | 191 (60.8) | 103 (62.0) | 10 (62.5) |
| Prefers patient (vs. doctor or both) make final medical decision, No. (%) | | 133 (42.4) | 72 (43.4) | 6 (37.5) |

© 2023 Ganguli I et al. *JAMA Network Open.*

## eTable 8. Baseline Characteristics of Interviewed Physicians and Patients (continued)

| **Patient Characteristics** | | **All enrolled patients (n = 314)** | **Intervention group patients (n = 166)** | **Interviewed patients (n = 16)** |
|---|---|---|---|---|
| How often someone helps read health materials, No. (%) | All or some of the time | 49 (15.6) | 25 (15.1) | 3 (18.8) |
| | A little of the time | 78 (24.8) | 42 (25.3) | 3 (18.8) |
| | None of the time | 180 (57.3) | 95 (57.2) | 8 (50.0) |
| | Not applicable | 7 (2.2) | 4 (2.4) | 2 (12.5) |

Interview participants were recruited using maximum variation sampling was based on age, gender, race/ethnicity, and survey responses on knowledge, preferences, and conversation content.

* Other includes non-Hispanic mixed racial background, some other race, and prefer not to answer.

† Other includes gender variant/non-conforming and prefer not to answer.

‡ Other includes uninsured, worker's compensation, and UniCare GIC.

§ Years with PCP includes multiply-imputed values for 74 patients.

## eTable 9. Summary of Qualitative Patient Descriptions of Test Conversation During Study Visit

| | Number of times theme or sub-theme emerged in survey responses from each group | | |
|---|---|---|---|
| **Themes and sub-themes, with sample quotes from survey** | **Total (n = 68)*** | **Intervention group (n = 36)*** | **Control group (n = 32)*** |
| **Theme: Justifies lack of test conversation** | 14 | 7 | 7 |
| Believes tests are routine care<br>“There was no need for elaborate conversations about tests which I routinely receive at any physical” (Patient #93) | 11 | 7 | 4 |
| Discussed previously or already understood reason for tests<br>“We both knew [how] important it is. We didn't need to talk much about it” (#77) | 3 | 0 | 3 |
| **Theme: Reasons for getting tests** | 12 | 7 | 5 |
| Believes tests are routine care<br>“Part of the normal course of my care” (#179) | 5 | 3 | 2 |
| Due to symptoms or medical condition<br>“Tests resulted from description of symptoms I described” (#89) | 5 | 3 | 2 |
| Believes tests are low burden<br>“… so obviously pretty low on the cost/stress/discomfort scale” (#288) | 2 | 1 | 1 |
| Patient wanted tests<br>“I was very interested in my blood test. I wanted to see how my cholesterol was. I like to see how my blood results trend over the years. I am not concerned if my insurance does not cover it.” (#116) | 1 | 1 | 0 |
| Patient trusts doctor<br>“Even though she did not discuss the exact purpose of the tests, we have a long relationship and I trust her judgment” (#252) | 1 | 1 | 0 |
| **Theme: Description of test conversation** | 26 | 13 | 13 |
| Satisfied with discussion<br>“I was very satisfied with the discussion we had about testing and give my doctor all the credit for making me feel comfortable and part of the decision-making process” (#362) | 14 | 5 | 9 |
| Unsatisfied with discussion<br>“My doctor refused to do certain screening exams for me.” (#70) | 1 | 0 | 1 |
| Decided there was no need for testing and/or can watch and wait instead<br>“Prior testing showed no ulcer however the problem remains and is being followed. There was no need for additional testing” (#37) | 6 | 5 | 1 |
| Not all ordered tests were discussed<br>“Did not mention all tests that were eventually ordered.” (#138) | 1 | 0 | 1 |
| Discussed prior test results<br>“We reviewed the last tests that I did and the possibility of redoing some of them” (#218) | 7 | 5 | 2 |

Patient responses to the open-ended prompt in the post-study survey, “please add any other comments about how your doctor talked with you about medical tests at this visit,” were qualitatively analyzed for themes and sub-themes. Counts in table indicate how many patient responses reflected each theme.

* n represents number of patients in each group who wrote something in response to the prompt. Counts do not sum to total as sub-themes are not mutually exclusive.

## eReferences


1. Society of General Int Med - Routine physicals | Choosing Wisely [Internet]. 2013 [cited 2023 Mar 20]. Available from: https://www.choosingwisely.org/clinician-lists/society-general-internal-medicine-general-health-checks-for-asymptomatic-adults/

2. Fletcher G. Evidence-based approach to prevention. In: Elmore J, Givens J, editors. UpToDate [Internet]. Waltham, MA: UpToDate; 2023 [cited 2022 Apr 8]. Available from: uptodate.com

3. Testing Wisely [Internet]. Testing Wisely. [cited 2023 Mar 20]. Available from: https://www.testingwisely.com

4. How one medical checkup can snowball into a 'cascade' of tests, causing more harm than good. Washington Post [Internet]. 2020 Mar 25 [cited 2023 Mar 20]; Available from: https://www.washingtonpost.com/health/how-one-medical-checkup-can-snowball-into-a-cascade-of-tests-causing-more-harm-than-good/2020/01/03/0c8024fc-20eb-11ea-bed5-880264cc91a9_story.html

5. Rudin RS, Thakore N, Mulligan KL, Ganguli I. Addressing the Drivers of Medical Test Overuse and Cascades: User-Centered Design to Improve Patient–Doctor Communication. The Joint Commission Journal on Quality and Patient Safety. 2022 Apr 1;48(4):233–40.


© 2023 Ganguli I et al. *JAMA Network Open.*
